# Supplementary material for: VIVALDI-CT shaping care home COVID-19 testing policy: A pragmatic cluster randomised controlled trial of asymptomatic testing compared to standard care in care home staff
Source: PLoS One. 2025 Jul 2;20(7):e0324908. doi: 10.1371/journal.pone.0324908 (PMC12221029; doi:10.1371/journal.pone.0324908)
Supplement: S1 Table — (DOCX) [file pone.0324908.s003.docx]

**Table S1** Simplifications required to achieve convergence of analysis models.

|  | Model simplifications vs statistical analysis plan |
| --- | --- |
| *Primary outcome* | **Event count outcome** |
| IR of COVID-19 hospital admissions per 1kPY | Linear adjustment for calendar time and number of residents at baseline. |
| *Secondary outcomes* | **Event count outcome** |
| IR of all-cause hospital admissions per 1kPY | Linear adjustment for calendar time. |
| IR of COVID-19 mortality in residents per 1kPY | Poisson model without any adjustment variables. |
| IR of all-cause mortality in residents per 1kPY | No adjustment for calendar month and linear adjustment for number of residents at baseline. |
| Composite IR of COVID-19 hospital admissions and mortality per 1kPY | Poisson model with no adjustment for calendar month and linear adjustment for number of residents at baseline. |
| IR of SARS-CoV-2 infections in residents per 1kPY | Linear adjustment for calendar time. |
|  | **Binomial outcome** |
| Proportion of staff testing each week (%) | No adjustment for calendar time. |
| Prevalence of SARS-CoV-2 among staff who test each week (%) | Linear adjustment for calendar time. |
| Proportion of staff per home off sick each week (%) | Linear adjustment for calendar time and number of residents at baseline. |
| Proportion of all shifts filled by agency staff each week (%) | No adjustment for calendar time. |

IR, incidence rate; 1kPY, 1000 person-years.
