# Supplementary material for: VIVALDI-CT shaping care home COVID-19 testing policy: A pragmatic cluster randomised controlled trial of asymptomatic testing compared to standard care in care home staff
Source: PLoS One. 2025 Jul 2;20(7):e0324908. doi: 10.1371/journal.pone.0324908 (PMC12221029; doi:10.1371/journal.pone.0324908)
Supplement: S2 Table — (DOCX) [file pone.0324908.s004.docx]

**Table S2** Intervention effect estimates from mixed effects models without adjustment variables.

|  | **Intervention vs control** |
| --- | --- |
| *Primary outcome* | **IRR (95% CI, *P*)** |
| IR of COVID-19 hospital admissions per 1kPY**†** | 1.56 (0.60-4.08, 0.36) |
| *Secondary outcomes* | **IRR (95% CI, *P*)** |
| IR of all-cause hospital admissions per 1kPY**†** | 0.71 (0.52-0.97, 0.03) |
| IR of COVID-19 mortality in residents per 1kPY**†** | 0.64 (0.15-2.70, 0.54) |
| IR of all-cause mortality in residents per 1kPY**†** | 1.09 (0.80-1.48, 0.58) |
| Composite IR of COVID-19 hospital admissions and mortality per 1kPY**†** | 1.22 (0.50-2.99, 0.66) |
| IR of SARS-CoV-2 infections in residents per 1kPY**†** | 1.73 (0.42-7.01, 0.45) |
|  | **OR (95% CI, *P*)** |
| Proportion of staff testing each week (%) | 29.16 (11.55-73.61, <0.01) |
| Prevalence of SARS-CoV-2 among staff who test each week (%) | 0.25 (0.09-0.65, <0.01) |
| Proportion of staff per home off sick each week (%) | 0.96 (0.74-1.25, 0.77) |
| Proportion of all shifts filled by agency staff each week (%) | 0.73 (0.19-2.76, 0.64) |

IR, incidence rate; OR, odds ratio; 1kPY, 1000 person-years. †Statistical model summaries do not include data from first 2 weeks of trial participation at each site.
