# Supplementary material for: VIVALDI-CT shaping care home COVID-19 testing policy: A pragmatic cluster randomised controlled trial of asymptomatic testing compared to standard care in care home staff
Source: PLoS One. 2025 Jul 2;20(7):e0324908. doi: 10.1371/journal.pone.0324908 (PMC12221029; doi:10.1371/journal.pone.0324908)
Supplement: S1 Appendix — (DOCX) [file pone.0324908.s005.docx]

***Appendix S1***

***Dropped analyses***

The following sensitivity analyses were specified in the Statistical Analysis Plan, but were dropped due to the lower than expected sample size for the trial:

“We will define an implementation score based on the frequency and proportion of staff testing at each home based on data the homes provide, which may vary over time. In exploratory analysis we will assess whether the primary outcome is associated with this implementation score within the intervention arm and express the effect of the intervention relative to control arm for different levels of implementation. This analysis will be based on the same regression method as used for the primary analysis.

Using interaction terms, we will explore whether the effect of the intervention on the primary outcome differed between time periods defined by the national recommendations for testing in the routine care arm, should these change. To date, we would evaluate the periods before 3 April 2023 (symptomatic testing in residents and staff plus outbreak testing) and 3 April and beyond (symptomatic testing only in residents who are eligible for covid therapeutics + outbreak testing). To allow better consideration of the likely intervention impact across the whole care home sector we will also explore whether the intervention effect differs according to care home size, and other characteristics such as proportion of temporary staff.”

***Comparison of primary outcome event count by data source***

|  | Count of Primary Outcome events [*n*] | |
| --- | --- | --- |
|  | Control | Intervention |
| *Individual-level admission events uploaded to COVID-19 Datastore by sites/provider* | 0 | 1 |
| *Weekly aggregate data on hospital admission events from sites/provider* | 1 | 7 |
| *Routine data: +ve test within 1 week prior to hospital admission or relevant ICD10 code anywhere in admission record* | 9 | 13 |
| *Routine data: +ve test within 1 week prior to hospital admission* | 3 | 7 |
| *Routine data: relevant COVID ICD10 code anywhere in admission record* | 8 | 13 |
| *Routine data: Primary COVID ICD10 code in admission record* | 3 | 4 |
| *Routine data: Secondary COVID ICD10 code in admission record* | 5 | 9 |

The single individual-level admission record that was uploaded by a care home for the purpose of trial data collection is consistent with the aggregate data for that week. This individual also has routine data records for a hospital admission with a primary ICD10 code for COVID-19 and a preceding positive SARS-CoV-2 test logged.

For the weekly aggregate data, the total of eight events comprises 3 weeks with one event reported and 1 week with five events reported. For the 3 weeks with one event, in two of these there is a corresponding single hospital admission recorded in the routine data (both primary cause), and in one there are two hospital admissions in the routine data (one primary + uploaded by site and one secondary). The week with five events reported was in June and does not correspond to any routinely collected hospital admission events.

For context, with a total of 22 primary outcome events the predicted 6-month cumulative incidence is 0.93%, in comparison to an assumed control arm cumulative incidence of 3.0% for the sample size calculations (with assumed average of 2.45% across the two trial arms).
