# Supplementary material for: VIVALDI-CT shaping care home COVID-19 testing policy: A pragmatic cluster randomised controlled trial of asymptomatic testing compared to standard care in care home staff
Source: PLoS One. 2025 Jul 2;20(7):e0324908. doi: 10.1371/journal.pone.0324908 (PMC12221029; doi:10.1371/journal.pone.0324908)

VIVALDI-CT

Shaping care home COVID-19 testing policy: A pragmatic cluster randomised controlled trial of an intervention to promote regular, asymptomatic testing in care home staff.

Clinical Trial Protocol

Version	v1.0
Date	25 October 2022
Sponsor	University College London
R&D #	151687
CCTU Trial Adoption Group #	CTU/2022/405
Trial registration	[ClinicalTrials.gov and identifier]
REC #	[22/LO/0846]
CAG #	[22/CAG/0165]

Authorisation: Chief Investigators

Name	Prof Laura Shallcross
Role	Chief Investigator

Signatures/date

Authorisation: Sponsor

Name	Prof Nick Freemantle
Role	Director, UCL CCTU

Signature/date

Authorisation: Senior Operations Staff

Name	Mr James Blackstone
Role	Clinical Project Manager, UCL CCTU

Signature/date

Authorisation: Senior Statistician

Name	Prof Andrew Copas
Role	Professor of Trials in Global Health, UCL IGH

Signature/date

Authorisation: Senior Health Economist

Name	Dr Catherine Henderson
Role	Assistant Professorial Research Fellow, LSE

Signature/date

Authorisation: Senior Qualitative Researcher

Name	Prof Paul Flowers
Role	Chair in Health Change, University of Strathclyde

Signature/ date

Table of Contents

Table of Contents 3

General information 8

Sponsor 8

Funding 8

Trial Registration 8

Trial Administration 8

Coordinating Centre 9

Structured trial summary 10

Roles and responsibilities 13

Protocol contributors 13

Role of trial sponsor and funders 13

Trial Team 13

Trial Management Group 14

Trial Steering Committee 15

Data Monitoring and Ethics Committee 15

Trial Diagram 16

Abbreviations 17

Glossary 18

1 Introduction 19

1.1 Background and rationale 19

1.1.1 Explanation for choice of comparator 22

1.2 Objectives 23

1.3 Trial Design 23

1.4 Risk Benefit Assessment 23

2 Selections of Sites/Investigators 25

2.1 Site Selection 25

2.1.1 Study Setting 25

2.1.2 Site/Investigator Eligibility Criteria 25

2.1.2.1 Principal Investigator’s (PI) Qualifications and Agreements 26

2.1.2.2 Resourcing at site 26

2.2 Site approval and activation 26

3 Selection of Participants 27

3.2 Participant Exclusion Criteria 27

3.3 Recruitment 27
3.4 Identification of staff, other participants and consent 28
3.5 Care home baseline data collection 29
3.6 Randomisation of sites 30
4 Trial Intervention 30
4.1 Introduction 30
4.2 Arm A 32
4.2.1 LFD testing for Covid-19 with sickness support payment 32
4.3 Arm B 33
4.3.1 Usual Care 33
4.4 Interruptions and Discontinuations 33
4.5 Concomitant Care 34
4.6 Blinding Considerations 34
4.7 Protocol Intervention Discontinuation 34
4.8 Compliance and Adherence 34
4.8.1 Withdrawal of Consent/assent 34
4.9 Eligibility Criteria for Individuals Performing the Interventions 35
5 Assessments & Follow-Up 35
5.1 Outcomes 35
5.1.1 Primary Outcome 35
5.1.2 Secondary Outcomes 35
5.1.3 Economic Outcomes (WP4) 36
5.1.4 Exploratory Analyses (WP3B) 36
5.2 Data sources 37
5.3 Trial Pathway 39
5.4 Post-trial Care 40
6 Safety reporting 41
6.1 Definitions 41
6.2 Adverse Events 42
6.3 Investigator responsibilities 42
6.3.1 Investigator Assessment 42
6.3.1.1 Seriousness 42
6.3.1.2 Causality 42
6.3.1.3 Severity or Grading of Adverse Events 43

6.3.1.4 Expectedness 44
6.3.2 Notifications 44
6.3.2.1 Notifications by the Investigator to CCTU 44
6.4 CCTU responsibilities 45
6.4.1 Urgent Safety Measures 45
7 Quality Assurance & Control 45
7.1 Risk Assessment 45
7.2 Central Monitoring at CCTU 46
7.3 Monitoring 46
7.3.1 Direct access to Participant Records 46
7.3.2 Confidentiality 46
7.4 Source Data 47
7.5 Data Collection and Transfer Methods 47
7.6 Data Management 48
7.7 Data Storage 48
7.8 Data Archiving 48
7.9 Quality Issues 49
8 Statistical Considerations 49
8.1 Sample Size 49
8.2 Assignment of Intervention 49
8.2.1 Randomisation 49
8.2.2 Sequence generation 50
8.2.3 Blinding 50
8.3 Statistical Methods 50
8.3.1 Statistical Analysis Plan 50
8.3.2 Interim Analyses 50
8.3.3 Statistical Methods – Overview 50
8.3.4 Statistical Methods – Coding of outcomes 51
8.3.5 Additional Analyses - Subgroup 51
8.3.6 Additional Analyses - Adjusted 51
8.3.7 Analysis Population and Missing Data 51
9 Economic Evaluations 51
9.1 Economic Analysis 51
9.2 Within-trial analysis 52

9.2.1 Outcomes 52
9.2.1.1 Resource Use 52
9.2.1.2 Health related Quality of life 53
9.2.2 Cost Data 53
9.2.2.1 Cost of the VIVALDI-CT intervention 53
9.2.2.2 Cost of Healthcare resource use 53
9.2.3 QALYs 54
9.2.4 Analysis results 54
9.2.4.1 Primary analysis 54
9.2.4.2 Missing data 54
9.2.4.3 Sensitivity analysis 54
9.2.4.4 Secondary analyses 54
9.3 Modelling analysis 54
10 Regulatory & Ethical Issues 55
10.1 Compliance 55
10.1.1 Regulatory Compliance 55
10.1.2 Site Compliance 55
10.1.3 Data Collection & Retention 55
10.2 Ethical Approvals 55
10.2.1 Ethical Considerations 55
10.2.2 Ethics Committee Approval 56
10.3 Competent Authority Approvals 56
10.4 Other Approvals 56
10.5 Trial Closure 57
11 Indemnity 57
12 Finance 57
13.1 Trial Management Group 58
13.2 Programme Steering Committee 58
13.3 Data Monitoring and Ethics Committee 58
13.4 Trial Sponsor 58
14 Patient & Public Involvement 58
14.1 Potential Impact of PPI 59
14.2 Identifying PPI Contributors 59
15 Publication & Dissemination of Results 60

15.1 Publication Policy 60
15.1.1 Trial Results 60
15.1.2 Authorship 60
15.1.3 Reproducible Research 60
16 Data Sharing 60
17 Process Evaluation (WP3A) 61
17.1 Background 61
17.2 Aims61
17.3 Objectives 61
17.4 Process Evaluation Outputs 61
17.5 Study Design 61
17.6 Theoretical framework 61
17.7 Sample identification and recruitment 61
17.8 Overview of Data Collection 62
17.9 Qualitative Data Analysis 62
17.10 Reporting and Dissemination 62
18 Protocol Amendments 62
References 63
Appendix A: VIVALDI-CT outbreak escalation decision algorithm 68

General information

This document was constructed using the Comprehensive Clinical Trials Unit (CCTU) at UCL Protocol template Version 6. It describes the VIVALDI-CT trial, sponsored and co-ordinated by CCTU.

It provides information about procedures for entering participants into the trial, and provides sufficient detail to enable: an understanding of the background, rationale, objectives, trial population, intervention, methods, statistical analyses, cost-effectiveness analyses, ethical considerations, dissemination plans and administration of the trial; replication of key aspects of trial methods and conduct; and appraisal of the trial's scientific and ethical rigour from the time of ethics approval through to dissemination of the results. The protocol should not be used as an aide-memoire or guide for the treatment of other patients. Every care has been taken in drafting this protocol, but corrections or amendments may be necessary. These will be circulated to registered investigators in the trial. Sites entering participants for the first time should confirm they have the correct version through a member of the trial team at CCTU.

CCTU supports the commitment that its trials adhere to the SPIRIT guidelines. As such, the protocol template is based on an adaptation of the Medical Research Council CTU protocol template and the Standard Protocol Items: Recommendations for Interventional Trials (SPIRIT) 2013 Statement for protocols of clinical trials.[1] The SPIRIT Statement Explanation and Elaboration document[2] can be referred to, or a member of CCTU Protocol Review Committee can be contacted for further detail about specific items.

Sponsor

UCL is the trial sponsor and has delegated responsibility for the overall management of the VIVALDI-CT trial to CCTU. Queries relating to UCL sponsorship of this trial should be addressed to the CCTU Director or via the Trial Team.

Funding

VIVALDI-CT is fully funded by an NIHR Health and Social Care Delivery Research (HSDR) Programme number [154310]. Costs associated with SARS-CoV-2 testing including support payments for care home staff will be funded by the UK Health Security Agency (UKHSA).

Trial Registration

This trial has been registered with the ClinicalTrials.gov Clinical Trials Register, where it is identified as [insert info].

Trial Administration

Please direct all queries to the Trial Manager at UCL CCTU in the first instance; clinical queries will be passed to the Chief Investigator by the Trial Manager.

Coordinating Centre

Comprehensive Clincial Trials Unit at UCL (UCL CCTU)
Institute of Clinical Trials & Methodology
2nd Floor, 90 High Holborn
London
WC1V 6LJ
UK
cctu.vivaldi@ucl.ac.uk

Structured trial summary

Acronym or short title	VIVALDI-CT
Scientific Title	Shaping care home COVID-19 testing policy: A pragmatic cluster randomised controlled trial of an intervention to promote regular, asymptomatic testing in care home staff.
CCTU Trial Adoption Group #	CTU/2022/405
Sponsor R&D ID #	151687
REC #	22/LO/0846
CAG #	22/CAG/0165
IRAS #	320847
Primary Registry and Trial Identifying Number	ClinicalTrials.gov
Date of Registration in Primary Registry	[Date when trial was officially registered in the primary registry.]
Secondary Identifying Numbers	NIHR UKCRN Portfolio:
Source of Monetary or Material Support	NIHR Health and Social Care Delivery Research (HSDR) Programme number 154310.
Sponsor	University College London with sponsor responsibilities delegated to CCTU, UCL.
Contact for Public Queries	ctu.enquiries@ucl.ac.uk
Contact for Scientific Queries	
Countries of Recruitment	UK: England
Health Condition(s) or Problem(s) Studied	To estimate the effectiveness, cost-effectiveness and feasibility of regular asymptomatic testing for SARS-CoV-2 in care home staff to reduce the impact of SARS-CoV-2 in care homes
Intervention(s)	**Intervention:** regular asymptomatic testing of care home staff for Covid-19 using Lateral Flow Devices (LFDs) combined with support payments for sickness absence<br><br>**Control**: Covid-19 testing policy for care home staff that is in place nationally at the time of trial operation.
Key Inclusion and Exclusion Criteria	**Inclusion Criteria:**<br>Only care home staff are eligible to participate in the testing intervention. This includes temporary (agency) staff with no restrictions i.e. catering staff, administrative staff, maintenance staff, in addition to those in a resident-facing role.

	All care home staff, residents, visitors and relatives are eligible to participate in interviews undertaken as part of the process evaluation.<br><br>All care home residents at participating home are eligible for data collection and analysis of the outcomes specified.<br><br>**Exclusion Criteria:**<br>Staff who visit the care home to provide care but are not employed by the care home e.g. GPs, health visitors are not eligible to take part in either the interviews or the testing intervention.
Study Type	VIVALDI-CT is a UK multi-centre, open-label, interventional, cluster randomised controlled, phase III trial
Study setting	Residential and/or nursing homes providing care to adults aged >65 years
Planned Date of First Enrolment	October 2022
Target Sample Size	280 care homes (the subjects of the trial are the care homes).
Trial Duration	1 year
Primary Outcome	• Number of COVID-19 related hospital admissions in residents.
Key Secondary Outcomes<br>*See 5.1.2 for full list*	• Incidence of hospital admissions (all-cause) in residents for non-elective care;<br>• Incidence of COVID-associated mortality in residents;<br>• Incidence of all-cause mortality in residents;<br>• Testing uptake in staff;<br>• Prevalence of COVID-19 among staff and residents who test;<br>• Outbreaks (incidence and duration);<br>• Testing metrics e.g. staff time taken to conduct the test at work, costs per test
Process Evaluation Outcomes	• Impact of testing on resident, staff and visitors assessed via Social Care related Quality of Life (SCRQoL)<br>• Feasibility / acceptability of the testing intervention

	• Fidelity/adaption of testing and payment<br>• Support for intervention mechanisms and content

Roles and responsibilities

These membership lists are correct at the time of writing; please see terms of reference documentation in the TMF for current lists.

Protocol contributors

Name	Affiliation	Role
Prof Laura Shallcross	UCL IHI	Chief Investigator
Prof Paul Flowers	University of Strathclyde	Senior Qualitative Researcher
Prof Andrew Copas	UCL IGH	Oversight Statistician
Dr Catherine Henderson	LSE CPEC	Senior Health Economist
Prof Jackie Cassell	Brighton & Sussex Medical School & UKHSA	Public health consultant leading analysis of SCRQoL
Dr Natalie Adams	UCL IHI	Speciality registrar in Public Health
Mr James Blackstone	UCL CCTU	Clinical Project Manager
Prof Nick Freemantle	UCL CCTU	Co-Investigator / Senior Statistician / Sponsor Representative
Dr Oliver Stirrup	UCL IGH	Trial Statistician
Dr Lara Goscé	UCL IGH	Mathematical Modeller
Natasha Southall	Four Seasons Healthcare	PPIE lead
Professor Martyn Regan	University of Manchester & UKHSA	Interaction with UKHSA Adult Social Care policy
Professor Adam Gordon	University of Nottingham	Clinician – care for older adults
Professor Aparna Verma	University of Manchester	PPIE and dissemination
Professor Susan Hopkins	UKHSA	Translation of results into policy and practice

Role of trial sponsor and funders

Name	Role
University College London Comprehensive Clinical Trials Unit	Sponsor
Health and Social Care Delivery Research (HSDR) Programme	Funder
UK Health Security Agency (UKHSA)	Funder

Trial Team

Name	Affiliation	Role and responsibilities
Prof Laura Shallcross	UCL IHI	Co-Chief Investigator

Prof Paul Flowers	University of Strathclyde	Co-Chief Investigator & Senior Qualitative Researcher
Prof Andrew Copas	UCL IGH	Oversight Statistician
Dr Catherine Henderson	LSE CPEC	Senior Health Economist
TBC	LSE CPEC	Health Economist
TBC	University of Strathclyde	Qualitative Researcher 1 (Intervention acceptability)
TBC	Brighton & Sussex Medical School	Qualitative Researcher 2 (SCRQoL)
Dr Lara Goscé	UCL IGH	Mathematical Modeller
Mr James Blackstone	UCL CCTU	Clinical Project Manager
Prof Nick Freemantle	UCL CCTU	Co-Investigator / Senior Statistician / Sponsor Representative
TBC	UCL CCTU	Trial Manager
TBC	UCL CCTU	Data Manager
Dr Oliver Stirrup	UCL IGH	Trial Statistician
Dr Natalie Adams	UCL IHI	Speciality registrar in Public Health

Trial Management Group

Name	Affiliation	Role and responsibilities
Prof Laura Shallcross	UCL IHI	Chief Investigator
Prof Paul Flowers	University of Strathclyde	Co-Chief Investigator & Senior Qualitative Researcher
Prof Andrew Copas	UCL IGH	Oversight Statistician
Dr Catherine Henderson	LSE CPEC	Senior Health Economist
TBC	LSE CPEC	Health Economist
Dr Oliver Stirrup	UCL IGH	Trial Statistician
Prof Nick Freemantle	UCL CCTU	Co-Investigator / Senior Statistician / Sponsor Representative
Mr James Blackstone	UCL CCTU	Clinical Project Manager
Dr Natalie Adams	UCL IHI	Speciality registrar in Public Health
Prof Jackie Cassell	Brighton & Sussex Medical School & UKHSA	Public health consultant leading analysis of SCRQoL
Dr Lara Goscé	UCL IGH	Mathematical Modeller
Natasha Southall	Four Seasons Healthcare	PPIE lead
Professor Martyn Regan	University of Manchester & UKHSA	Interaction with UKHSA Adult Social Care policy

Professor Adam Gordon	University of Nottingham	Clinician – care for older adults
Professor Aparna Verma	University of Manchester	PPIE and dissemination
Professor Susan Hopkins	UKHSA	Translation of results into policy and practice

Trial Steering Committee

Name	Affiliation	Role
TBC		Chair (independent)
TBC		Statistician (independent)
TBC		TBC (independent)
TBC		PPI (independent)

Data Monitoring and Ethics Committee

Name	Affiliation	Role
TBC		Chair (independent)
TBC		Statistician (independent)
TBC		Clinician (independent)

Trial Diagram

Figure 1: Flow diagram of cluster randomised control trial design

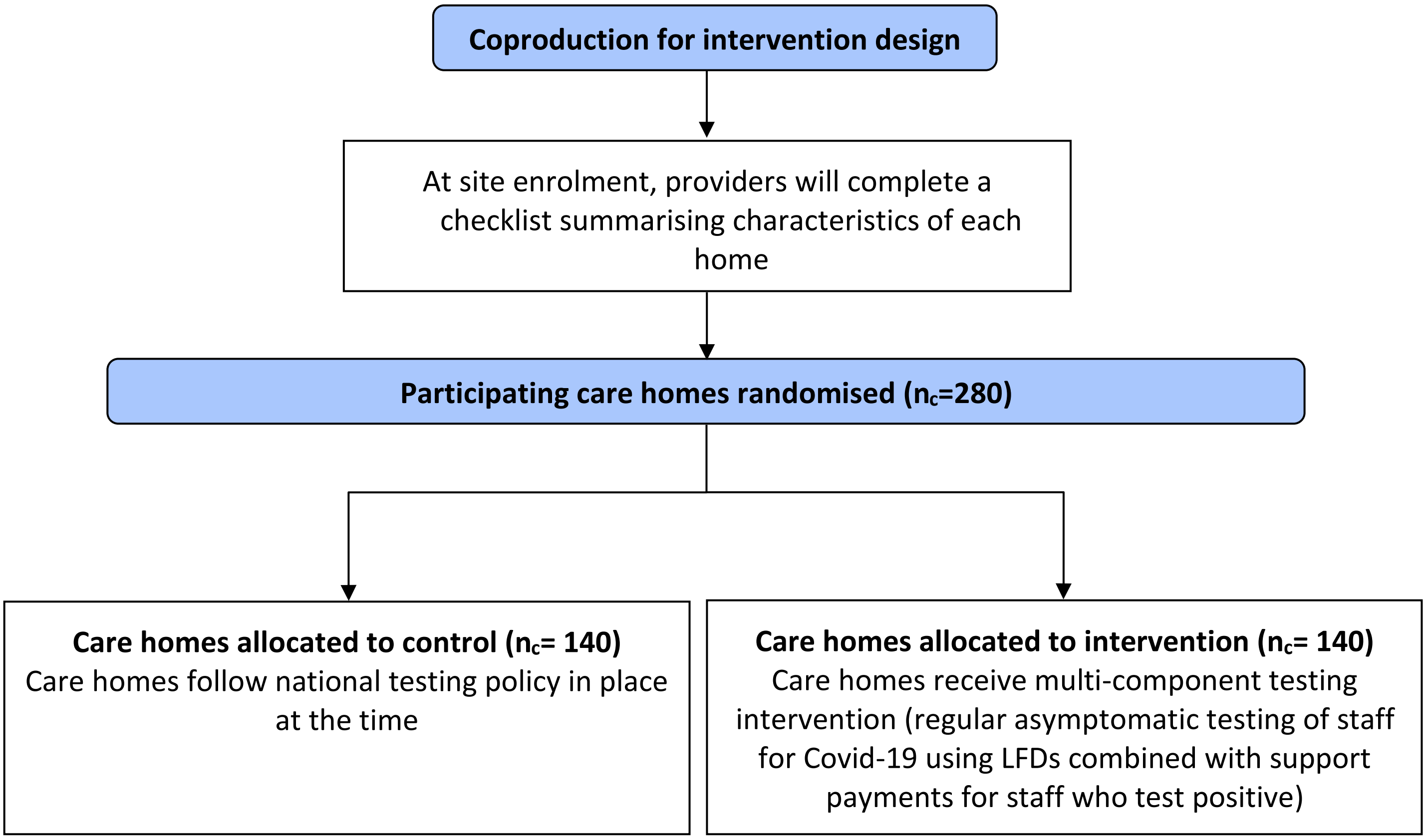

Abbreviations

AE	Adverse Event
AR	Adverse Reaction
CAG	Confidentiality Advisory Group
CEA	Cost-Effectiveness Analysis
CEAC	Cost-Effectiveness Acceptability Curve
CEP	Cost-effectiveness Plan
CCTU	Comprehensive Clinical Trials Unit at UCL
CI	Chief Investigator
CRF	Case Report Form
CTCAE	Common Terminology Criteria for Adverse Events
DHSC	UK Department of Health and Social Care
DMEC	Data Monitoring and Ethics Committee
EC	Ethics Committee
EDC	Electronic Data Capture
EHR	Electronic Health Record
GCP	Good Clinical Practice
GP	General Practitioner
HE	Health Economist
HEAP	Health Economics Analysis Plan
HRA	Health Research Authority
ICER	Incremental Cost-Effectiveness Ratio
ICH	International Conference on Harmonisation
ICF	Informed Consent Form
IDMC	Independent Data Monitoring Committee
IRAS	Integrated Research Application System
ISF	Investigator Site File
ISRCTN	International Standard Randomised Controlled Trial Number
ITT	Intention to Treat
LFD	Lateral Flow Device
MRC	Medical Research Council

NHS	National Health Service
NHSE	NHS England
NIHR	National Institute for Health and Care Research
NPT	Normalisation Process Theory
PI	Principal Investigator
PIN	Participant Identification Number
PIS	Participant Information Sheet
PPI	Patient and Public Involvement
PSS	Personal Social Services
QA	Quality Assurance
QALY	Quality-Adjusted Life-Years
QC	Quality Control
QMMP	Quality Management and Monitoring Plan
QOL	Quality of Life
RCA	Root Cause Analysis
R&D	Research and Development
REC	Research Ethics Committee
SAE	Serious Adverse Event
SAP	Statistical Analysis Plan
SAR	Serious Adverse Reaction
SOP	Standard Operating Procedure
TMF	Trial Master File
TMG	Trial Management Group
TSC	Trial Steering Committee
ToR	Terms of Reference
UCL	University College London
UKHSA	UK Health Security Agency

Glossary

- **Case Report Form** - an electronic document designed to record all events within the study protocol required on each study subject.
- **COVID-19** - the disease caused by the novel SARS-CoV-2 virus.
- **Care home** - in the UK, care homes provide 'accommodation, together with nursing or personal care, for persons who are or have been ill, who have or have had a mental disorder, who are disabled or infirm, or are or have been dependent on alcohol or drugs.[3] They include homes with and without 24 h onsite nursing staff, known as residential and nursing homes, respectively.[4]
- **Nursing home** - care home with 24 h onsite nursing staff
- **Provider -** UK care home groups
- **Residential home** - care home without 24 h onsite nursing staff

1 Introduction

1.1 Background and rationale

In England, approximately 380,000 people (4% of > 65 year olds) live in 11,000 care homes for older adults. The majority of care home residents (hereafter 'residents') are aged > 85 years, at least two-thirds live with dementia, and over half die within 12 months of admission to a care home.[5,6] Residents worldwide have experienced among the highest rates of COVID-19 mortality and morbidity,[7] and in England they have also been subject to particularly strict and lengthy lock-down measures. Prolonged use of COVID-19 restrictions (e.g. social isolation, visitor restrictions) has had a devastating impact on residents' well-being, and their physical and mental health, for example depriving them of contact with family members in their final weeks of life.[8]

A range of public health disease control measures was deployed rapidly and simultaneously in care homes in the first wave of the pandemic to reduce the spread of infection, making it difficult to discern the impact of any individual measure. There have been no interventional studies of non-pharmaceutical control measures to reduce COVID-19 infection and related outcomes in care homes, but a Cochrane rapid review (published in September 2021) identified 11 observational and 11 modelling studies, all of which were from high-income countries.[9] The review grouped interventions into entry regulations (e.g. preventing visitors from entering the care home), contact regulating and transmission-reducing measures (e.g. Personal Protective Equipment, PPE), surveillance measures (symptomatic and asymptomatic testing), and outbreak control measures. Across these domains the quality of evidence was poor. In England, many of these control measures, such as preventing visitors from entering the care home were withdrawn at the earliest opportunity because they caused significant harm.

The two measures that have been used consistently to reduce the spread of infection in care homes throughout the pandemic are face masks and COVID-19 testing. As of 16th September 2022, symptomatic testing and whole care home testing following the detection of outbreaks is still in place for care home residents and staff; however, UKHSA guidance providing for asymptomatic testing for staff was withdrawn on 31st August 2022.

Evidence on the use of testing to reduce transmission of COVID-19 in care homes

Testing has been used in three ways to reduce transmission: 1) test people with symptoms, 2) test during outbreaks to reduce their duration and severity and 3) regular, asymptomatic testing. In England, by 6th September 2020 regular COVID-19 testing using Polymerase Chain Reaction (PCR) was well-established for residents (monthly) and staff (weekly) in addition to symptomatic testing and outbreak testing. Twice weekly testing for staff with Lateral Flow Devices (LFDs) was introduced on 23rd December 2020, in response to the emergence of the alpha variant. Regular testing for residents ceased at the end of March 2022 whereas twice

weekly asymptomatic testing for staff continued until 31st August, 2022, with the expectation that it may be reintroduced if rates of infection increase rapidly in the community and/or further variants emerge.

The Cochrane review cited above identified two observational studies,[10,11] which evaluated the effectiveness of different testing strategies for COVID-19 in care homes. There is low certainty evidence from one observational study,[10] and from four modelling studies,[12–15] that regular asymptomatic testing may reduce the number of infections, and one modelling study suggests it might also reduce hospitalisations (moderate certainty evidence), and/or deaths (low certainty evidence).[15] Evidence from one observational study and one modelling study suggests symptomatic testing may reduce the number of infections,[11,14] and probability of outbreaks (low certainty evidence), compared to no testing. However, both observational studies in the review were small (< 30 homes) and undertaken in Georgia, USA, before vaccination was introduced.

The majority of the modelling studies have focused on the test itself (e.g. PCR versus LFD), testees (residents, staff or both), or how often to test rather than the impact of testing per se. We have not considered PCR testing here because of its higher cost (c. £13/test) relative to LFDs (c. £1.50)/test. Similarly, we have not considered the use of regular testing in residents because of ethical concerns (testing may not directly benefit individual residents, and unlike staff they may find it difficult to opt out of testing). Although modelling studies suggest that higher testing frequency predicts lower rates of infection, we are constrained by costs and the practicalities of testing, so the maximum testing frequency that will be considered in this proposal is daily pre-shift testing for staff.

The Cochrane review's heavy reliance on modelling studies to explore the likely effectiveness of testing brings particular limitations when considering real world asymptomatic testing. There have been no evaluations of the cost-effectiveness of testing, and to our knowledge no studies have assessed the broader impacts of testing or outbreak restrictions on social care related quality of life, which remains poorly understood. It is also not possible to understand the implementation of testing in care homes because compliance has not been examined. Equally, the potential role of behaviour change interventions such as staff sickness pay in improving compliance with any given testing approach has not been explored.

Evidence on strategies to support compliance with regular testing in care homes

DHSC and UKHSA provided accessible guidance and on-line training (e.g. webinars) to support compliance with testing. In the UK, it is probable that compliance with regular testing has been driven by national policies to incentivise testing, including with financial support (e.g. Adult Social Care Rapid Testing Fund introduced in January 2021, Infection Control Fund introduced May 2020).[16,17] However, there are relatively few published studies that examined the influences on compliance with asymptomatic testing in care homes. We

conducted a rapid systematic review, spanning January 2020 to July 2022. It highlighted 14 international papers,[18–31] published in English. No studies used an experimental design and none reported, or evaluated, interventions designed to improve compliance with COVID testing. The 14 papers used a range of designs (e.g., qualitative, cross-sectional quantitative, consensus building). Together these studies highlight the *multi-levelled* factors that have shaped compliance with COVID testing in care homes.

The literature highlights the centrality of a series of *high-level issues* including national and regional policy and guidance, the financial models supporting testing, test availability and the establishment of effective partnerships between care homes, public health and health services. At the *level of care homes*, the literature also shows a range of important factors including financial aspects such as reimbursement for staff absences, the need for consistent and understandable guidance, the importance of planning and protocols, the active involvement of those being tested, and shared beliefs in relative value of testing to reduce COVID transmission. The literature also highlights the administrative burden of testing, dilemmas associated with strained staff capacity and the care home estate, risks of reputational damage, care home heterogeneity across the sector, and the poor quality of training. At *the individual level* the literature suggests testing compliance is shaped by its endorsement by management, support from peers, professional pride, beliefs in the efficacy, and particular added value of testing, and beliefs that the benefits of testing outweigh the varied costs. Equally, compliance with testing was constrained by concerns about its financial implications and work load, questions of the efficacy of test kits, poor communication about testing with management, inadequate training and confidence as well as the timing of staff testing.

Justification for the trial

Care homes provide residential and nursing care to the most vulnerable members of society. The care home workforce, which is primarily female, ethnically diverse and poorly paid is depleted and overstretched with an estimated 9% of posts in adult social care in England currently vacant.[32] Staff turnover is high: the average turnover rate for those with less than one years' experience in the sector was 44%, compared to 21% for those with 20 or more years of experience.[33]

More testing for COVID-19 has taken place in care homes than in any other setting (59 million PCR tests and 197 million LFDs since the start of the pandemic).[34] home staff were the only group for whom vaccination was mandated and they have been tested at least once per week since June 2020 until September 2022. Testing causes discomfort, adds significantly to staff members' workload, and a positive test culminates in self-isolation and loss of income because most providers do not fund full sickness pay. Regular testing in staff may also impact negatively on residents, by increasing staff absence which impacts on the delivery of care, and increasing detection of outbreaks leading to cancellation of communal activities.

At the start of the pandemic, policymakers' priority was to reduce deaths, severe illness and hospital admissions. The strong epidemiological link between rates of infection in the community and risk of outbreaks and severe outcomes in residents justified the use of testing to prevent ingress of infection from the community.[34] However, most residents are now fully vaccinated (90% have received 4th dose (3rd booster) vaccination with 5th dose currently being rolled out),[34] and many have been previously infected,[35] which substantially reduces their risk of COVID-19 related severe outcomes. Providers' main concern is that regular testing will increase the risk of care home closures due to outbreaks, impacting on business continuity (loss of income from new admissions) and residents' wellbeing (e.g. restrictions on visits). Based on current evidence, it is unclear whether regular testing will increase or reduce the frequency of outbreaks. Two and a half years into the pandemic, there remains a lack of evidence on whether the benefits of regular testing for COVID-19 outweigh its harms, and if so, under which scenarios. Also, there have been no attempts in the literature to consolidate the considerable expertise and learning on how to ensure compliance with testing in this setting. From a policy perspective, the key question remains over the thresholds for turning testing 'on' and 'off' in response to varying levels of 'COVID-19 threat' (e.g. high/low levels of infection in the community; emergence of novel COVID-19 variants).

We posit that the best approach to address these questions is through a randomised clinical trial. Randomisation overcomes the problem that care homes are extremely heterogeneous for example in terms of their resident population, care provision, and uptake of control measures such as vaccination in staff, which limits the conclusions that can be drawn from observational studies. The trial could be established quickly because 1) infrastructure for testing and data collection is already in place,[36] 2) care homes will not require training to deliver asymptomatic testing, and 3) implementation strategies included in the intervention (e.g. support payments for staff who test positive) are likely to incentivise trial participation.

We have previously demonstrated that it is feasible to set-up and deliver large scale studies in care homes on short time-scales by working with large providers.[37–40] Replicating this approach in a trial would represent a new, agile model for the rapid delivery of policy-relevant research, and appeal to care home stakeholders who are frustrated by traditional research timelines. Finally, UKHSA have agreed to fund the substantial costs of testing and support payments for staff until spring 2023.

In summary, this trial will investigate whether continued use of regular asymptomatic testing in staff is feasible, effective and cost-effective strategy to reduce the impact of COVID-19 in care homes. Findings will shape policy across the UK for COVID-19, and inform wider strategies to prevent other respiratory viruses in care homes, such as influenza.

1.1.1 Explanation for choice of comparator

Care homes in the control arm will be subject to the testing policy that is in place nationally at the time of the trial. This is because it would be unethical to withhold staff testing in control

homes if testing is recommended as part of national public health policy, given the vulnerability of care home residents to severe outcomes following Covid-19 infection.

Regular testing is not currently in place nationally; however, if this policy is introduced during the trial we anticipate that testing uptake will still be substantially lower in the control arm compared to the intervention arm. This is because staff in intervention homes will receive support payments to self-isolate whereas those in control homes will not. Lack of support payments is likely to strongly disincentivise staff members' to comply with regular testing. We aim to capture data on the uptake of testing direct from care homes (using routine data). Compliance with testing will also be explored in the process evaluation.

1.2 Objectives

To evaluate the effectiveness, cost-effectiveness, feasibility and sustainability and impacts on residents, staff and providers of regular, asymptomatic testing of care home staff for COVID-19.

The objectives will be delivered through a series of interlinked work-packages (WPs):

- To coproduce a sustainable COVID-19 testing intervention for care home staff (WP1)
- To evaluate the effectiveness of the testing intervention compared to the recommended testing protocol that is in place at the time in a pragmatic cluster randomised trial (WP2)
- To conduct a mixed methods process evaluation and exploratory analysis to:
  - Establish intervention acceptability, fidelity/adaptability, support for intervention mechanisms/content (WP3A)
  - Explore the impact of testing and outbreaks on social care related quality of life (WP3B)
- To evaluate the costs and benefits of the testing intervention from the perspective of the NHS, providers, residents, families and staff (WP4A)
- To model costs and benefits of the testing intervention under different scenarios (WP4B)
- To coproduce recommendations on the use of regular testing for policymakers (WP5)

1.3 Trial Design

VIVALDI-CT is a UK multi-centre, open label, cluster randomised controlled, phase III/IV superiority trial. Sites will be randomised on a 1:1 ratio.

1.4 Risk Benefit Assessment

The trial aims to quantify the benefits and harms of regular asymptomatic testing in care home staff to inform policy. The rationale for regular asymptomatic testing is that it may reduce the risk of severe disease in residents and the frequency/severity of outbreaks.

However, regular testing has negative consequences for staff, residents and providers and the benefits of regular testing depend on the likelihood of severe outcomes following Covid-19 infections, which are much reduced compared to the start of the pandemic due to high rates of vaccination and natural immunity in residents. There is no data from trials, and very limited data from modelling and observational studies on the impact of regular asymptomatic testing of staff to prevent infection and related healthcare outcomes in residents and there is a complete absence of data on the broader impacts of testing on residents, families, staff and providers.

There are three groups that could experience adverse effects as a result of this trial:

1. Care providers: The trial could lead to loss of income for providers if asymptomatic testing results in increased detection of low-risk cases leading to potentially unnecessary care home closures. Staff sickness and increased reliance on temporary staff will also increase provider costs. These risks have to be balanced against the potential benefits to residents and the wider health and social care system of reducing residents' risk of severe outcomes following infection, and preventing outbreaks through prompt detection and isolation of infected staff. The risk to providers that asymptomatic testing will increase (unnecessary) care home closures is mitigated by the trial team who will risk assess asymptomatic cases that have been detected in staff in the intervention arm to avoid unnecessary escalation of low-risk cases to local public health teams (See Appendix A). The trial team includes a number of individuals who have trained in health protection and are thus qualified to undertake this role.
2. Care home staff: Increased detection of asymptomatic infection may have negative financial consequences for staff if it increases sickness absence and leads to loss of income. This risk is mitigated by provision of support payments to staff who test positive.
3. Care home residents: Increased staff sickness absence may reduce staffing ratios and the quality of care that is delivered to residents. The process evaluation and exploratory analyses (WP3A and WP3B) will explicitly address these issues, providing critical data to inform balanced decisions on the longer term use of asymptomatic testing in care homes. Data on rates of infection, care home closures and staff sickness absence will also be reviewed by the IDMC.
4. Finally, there is a risk of introducing infection into the care home when the study team conduct in person data collection as part of the process evaluation and exploratory analyses (although most data collection is virtual). This risk will be mitigated by complying with infection control protocols in place at the time of the study (including asymptomatic testing where appropriate), not attending if unwell and using virtual meetings where possible.

2 Selections of Sites/Investigators

2.1 Site Selection

The trial sponsor has overall responsibility for site and investigator selection and has delegated this role to CCTU. All sites will have their feasibility for participation in the trial reviewed and assessed in line with CCTU SOPs to ensure sufficient staffing and resource.

2.1.1 Study Setting

We will first approach the following UK care home groups ('Providers'), which already have established relationships with the co-applicants:

1. Four Seasons Healthcare (FSHC)
2. HC-One
3. Orders of St John Care Trust (OSJCT)

Additional providers may be approached to participate in the event of site activation/recruitment challenges.

In view of the timescales for trial delivery, and the need to streamline and centralise data collection we will partner with providers that manage at least 50 homes. Homes will be selected to capture diversity in care home size, population (nursing / residential / dementia care), ethnicity, geographical location, rural/urban and provider type (for profit / not for profit). However, it is not possible to recruit small, independent care home chains, which account for a large proportion of homes in the UK (in 2017, over 80% of care home providers only operated one home[41]), to participate in the trial, due to the trial timescales. However, in WP5 we aim to engage a large range of stakeholders including those from small independent care homes to specifically explore the translation of trial findings across the sector and the development of implementation plans. We will also use mathematical modelling (WP4B) to explore the application of our findings to different kinds of care homes, for example by considering care home size.

Providers that take part in the trial will each identify a project manager who will be responsible for liaising between the trial team, trial sites (primarily care home managers) and the provider's senior management team.

2.1.2 Site/Investigator Eligibility Criteria

To participate in the VIVALDI-CT trial, investigators and providers must fulfil a set of criteria that have been agreed by the Sponsor and VIVALDI-CT Trial Management Group (TMG) and that are defined below.

Eligibility criteria:

1. A named individual is willing and appropriate to take Principal Investigator responsibility on behalf of the provider.

2. The care home manager has consented for the home to participate.
3. Staff have access to training/resources to ensure the testing intervention can be delivered and collection of trial-specific data.
4. The provider has appropriate information governance systems in place to ensure they can submit confidential individual-level, electronic data on residents to NHSE at regular intervals.

Providers meeting eligibility criteria and that are accepted by the Sponsor/TMG as being suitable to recruit to the trial will be issued with the VIVALDI-CT document set so that an assessment of capacity and capability can be made. All providers will confirm capacity and capability and a virtual site initiation visit (SIV) for both their central team and the individual home leads will be performed prior to site activation.

2.1.2.1 Principal Investigator's (PI) Qualifications and Agreements

The Principal Investigator(s) must be willing to sign a Principal Investigator Declaration on behalf of the provider to comply with the trial protocol (confirming their specific roles and responsibilities relating to the trial, and that their site is willing and able to comply with the requirements of the trial). This includes confirmation of appropriate qualifications (provision of an up-to-date CV), familiarity with the appropriate use of the interventional equipment and agreement to comply with the principles of Good Clinical Practice (GCP). The PI must agree to permit monitoring and audit as necessary at the site, and to maintain documented evidence of staff who have been delegated significant trial related duties.

The PI will typically be the designated project manager for each care provider.

2.1.2.2 Resourcing at site

- The investigator should have sufficient time to conduct properly and complete the trial within the agreed trial period.
- The investigator should have available an adequate number of qualified staff and suitable facilities for the anticipated duration of the trial in order to conduct the trial properly and safely.
- The investigator should ensure that all persons assisting with the trial are adequately informed about the protocol, the intervention, and their trial-related duties and functions.
- The site should have sufficient data collection resources to allow prompt data return.

2.2 Site approval and activation

Provider training will be performed prior to the activation of each site and will include all processes for the trial including but not limited to protocol training, data management procedures, procedures for handling of the intervention, serious adverse event reporting procedures and frequency and expectations for monitoring visits. A log of Site Initiation Visit attendees will be kept in the Investigator Site File (ISF) as a record of participants present. The

Visit will occur via Videoconference as outlined in the VIVALDI-CT CCTU Quality Management and Monitoring Plan (QMMP).

The trial manager or delegate will notify the PI in writing of the plans for site activation. Sites will not be permitted to commence the testing intervention until a letter for activation has been issued by CCTU. On receipt of the signed Clinical Trial Site Agreement (including the signed PI Declaration), completed delegation of responsibilities log and staff contact details, the Trial Manager or delegate will complete the green light process and issue written confirmation of site activation to the site PI.

The site must conduct the trial in compliance with the protocol as agreed by the Sponsor and which was given favourable opinion by the Ethics Committee (EC). The PI or delegate must document and explain any deviation from the approved protocol and communicate this to the trial team at CCTU.

A list of activated sites may be obtained from the Trial Manager.

3 Selection of Participants

There will be **NO EXCEPTIONS** (waivers) to eligibility requirements at the time of trial entry.

3.1 Participant Inclusion Criteria

Only care home staff are eligible to participate in the testing intervention. This includes temporary (agency) staff with no restrictions i.e. catering staff, administrative staff, maintenance staff, in addition to those in a resident-facing role.

However, all care home staff, as well as residents, visitors and relatives are eligible to participate in interviews undertaken as part of the process evaluation.

All care home residents at participating homes are eligible for data collection and analysis of the outcomes specified.

3.2 Participant Exclusion Criteria

Visitors, residents and relatives are not eligible to take part in the testing intervention.

Staff who visit the care home to provide care but are not employed by the care home e.g. GPs, health visitors are not eligible to take part in either the interviews or the testing intervention.

3.3 Recruitment

Rather than recruiting individual care homes, we will first contact the senior management teams of providers that we have previously worked with in the Vivaldi study[37] to determine if they are interested in participating in the trial. Providers will be asked to nominate a study lead (Principal Investigator) who will supply a list of eligible care homes and confirm that the

care home manager has provided consent for each listed home to participate. The listed providers deliver care to >400 homes in England so we anticipate it should be feasible to recruit the required number of homes by partnering with them.

If we are unable to recruit sufficient homes from the Vivaldi network, we will work with provider representative organisations (e.g. National Care Forum, Care England) to identify other large providers that are eligible for the trial. Our strategy of approaching provider senior management teams rather than care home managers is an efficient way to recruit large numbers of care homes. It also capitalises on providers' organisational infrastructure. In the intervention development phase (WP1) we will explore the use of financial incentives to encourage care home staff to participate in the trial. The precise format for these payments will be agreed in the coproduction workshops, but it is likely to involve a regular e.g. monthly contribution to participating care homes' staff fund, which is controlled by staff rather than providers. This will be in addition to provision of support payments for staff to self-isolate when unwell, see 4.2.

3.4 Identification of staff, other participants and consent

The eligibility criteria for staff participating in the trial are broad and will typically involve most staff routinely present at a participating care home. The PI (study project manager at each provider) will publicise REC-approved information about the trial to colleagues throughout the participating care homes in their group to raise awareness of clinical trial, its aims and what the logistics will involve. The trial team will support each PI by providing relevant REC approved, multi-modal materials as requested e.g. webinars, FAQs, videos.

Care home providers and home managers will be asked if their care home(s) are willing to participate in the trial, but individual-level consent for trial participation will not be sought from care home staff. Individual staff members will have the option to opt out of testing. The study will collect limited individual-level identifiable data from residents to ensure the primary outcome can be determined accurately, however it is not feasible to seek individual-level consent from residents for use of these data because many residents have cognitive impairment.

The study team already have experience of seeking permission from the HRA Confidentiality Advisory Group (CAG) to access confidential data on residents and staff to support the pandemic response to COVID-19 in care homes from the Vivaldi study. We will submit a new, related application to HRA CAG to seek a Section 251 exemption as a basis to legally hold these patient data for the duration of the trial.

Care home managers in the subset of homes that have been selected for qualitative data collection (focus groups or one to one interviews in WP3A) will be asked to disseminate recruitment materials to staff within the home via word of mouth, email or other routine modes of communication. Researchers can also deliver a brief presentation within routine staff meetings to advertise the study. According to care home manager preferences,

interested staff will then either liaise with the care home manager about their interest in participation, or respond directly to the research team. Upon receipt of staff contact details, interested staff will then be sent participant information sheets about the study, given the option to ask questions about the study, complete on-line consent forms and provide brief sociodemographic details to enable the study team to monitor total sample composition. The study team will then .arrange a mutually convenient time for data collection to take place. Within data collection (focus groups or one to one interviews), having checked on-line consent has already been given and after exploring any remaining unanswered questions raised by the PIS, the participants will be asked to also give recorded oral consent to participate. This consent will be recorded on the audio/video file and the researcher. Interviewees will be reimbursed for their time (vouchers).

As part of 3a we will also recruit residents (with capacity to consent), relatives or visitors to assist with the process evaluation through six focus groups. We will work with the a range of care home managers within the trial to offer proportionate yet inclusive participation. Care home managers will be asked to disseminate recruitment materials to selected residents, relatives or visitors via word of mouth, email or other routine modes of communication. According to care home manager preferences, interested parties will then either liaise with the care home manager about their interest in participation, or respond directly to the research team. Upon receipt of resident, relative or visitor contact details, interested participants will then be sent participant information sheets about the study, given the option to ask questions about the study, complete on-line consent forms and provide brief sociodemographic details to enable the study team to monitor total sample composition. The study team will then arrange a mutually convenient time for data collection to take place. Within data collection (focus groups or one to one interviews), having checked on-line consent has already been given and after exploring any remaining unanswered questions raised by the Participant Information Sheet, the participants will be asked to also give recorded oral consent to participate. This consent will be recorded on the audio/video file and the researcher. Interviewees will be reimbursed for their time (vouchers).

3.5 Care home baseline data collection

At site enrolment, providers will complete a checklist summarising characteristics of each participating care home. This will include:

- location of home (postcode)
- type of home (for profit/not for profit)
- size of chain (total number of homes owned/operated by provider in England)
- number of permanent staff and composition
- number of temporary staff
- number of residents
- type of care (nursing/residential/dementia)
- size of home (total number of beds/beds occupied)

- number of nursing/residential /dementia beds
- monthly bed occupancy in the prior 3 months
- number of residents funded by the local authority
- number of privately funded residents
- ethnicity of staff (broad categories not individual data)
- number of outbreaks in the last 3 months
- whether the home is closed to visitors
- disease control measures in place (e.g. cleaning frequency, cohorting etc.)
- vaccination uptake
- care quality (CQC rating)

3.6 Randomisation of sites

Restricted randomisation will be used to ensure balance on care home size, provider and region between the intervention and control arms. Further details are given in the 'Statistical considerations' section of this protocol.

The results of the randomisation process will be communicated to each provider's project manager. They will be responsible for informing care home managers whether they have been randomised to the control or intervention group. The trial team will draft an email / text that can be used to explain the process of randomisation to care home managers.

4 Trial Intervention

4.1 Introduction

Intervention Development: WP1

It is important to recognise that care providers and staff have extensive experience of using LFDs, which were introduced in care homes in November/December 2020. Staff have been tested for COVID-19 at least once per week since autumn 2020 until September 2022, and care home managers are highly knowledgeable about barriers to LFD testing and how to make it work in their setting. Staff have self-tested and tested residents, so the amount of training required in relation to the test itself is likely to be minimal. The greatest challenge for the trial is likely to be adherence to testing and ensuring that test results are recorded accurately.

An important aim of the trial is to develop a testing intervention that is sustainable and acceptable to care home stakeholders. It is therefore critical that the testing intervention is co-developed with providers, policymakers and care home staff, and considers the testing policy that is in place nationally at the time of the trial. Work to date (analysis of published literature, consultation with diverse UK care home managers, discussions with members of the National Care Forum) has suggested a series of potentially useful intervention components, which are adaptive to potential changes in national policy directly affecting the control arm. To further develop and finalise the intervention, we will hold a series of

workshops with care home stakeholders (e.g. home managers, staff, providers) and policymakers (including those with knowledge of testing logistics) to explore these intervention components. Using world cafe style methodology,[41] we will: 1) consolidate existing insights into routine testing gleaned through past experiences with the COVID-19 pandemic; 2) discuss the intervention prototype; and, 3) operationalise it in ways which are likely to be acceptable and appropriate within the sector. Data (transcripts, detailed notes, images) will be analysed thematically to further develop our initial programme theory specifying important elements of the context, key intervention components, their mechanisms, and their relation to a hierarchy of outcome measures (e.g. health, social and financial outcomes).

To recruit staff to intervention development workshops we will liaise with the National Care Forum and a range of care home managers. The latter will be asked to disseminate recruitment materials to staff within the home via word of mouth, email or other routine modes of communication. Researchers can also deliver a brief presentation within routine staff meetings to advertise the study. According to care home manager preferences, interested staff will then either liaise with the care home manager about their interest in participation, or respond directly to the research team. Upon receipt of staff contact details, interested staff will then be sent participant information sheets about the study, given the option to ask questions about the study, complete on-line consent forms and provide brief sociodemographic details to enable the study team to monitor total sample composition. The study team will then arrange a mutually convenient time for data collection to take place. Within data collection (focus groups or one to one interviews), having checked on-line consent has already been given and after exploring any remaining unanswered questions raised by the PIS, the participants will be asked to also give recorded oral consent to participate. This consent will be recorded on the audio/video file and the researcher. Interviewees will be reimbursed for their time (vouchers).

We will also recruit a single workshop with around eight residents (with capacity to consent), relatives, or visitors - to assist with intervention development. Again, we will work with the National Care Forum and a range of care home managers to offer proportionate yet inclusive participation. Care home managers will be asked to disseminate recruitment materials to selected residents, relatives or visitors within the home via word of mouth, email or other routine modes of communication. According to care home manager preferences, interested parties will then either liaise with the care home manager about their interest in participation, or respond directly to the research team. Upon receipt of resident, relative or visitor contact details, interested parties will then be sent participant information sheets about the study, given the option to ask questions about the study, complete on-line consent forms and provide brief sociodemographic details to enable the study team to monitor total sample composition. The study team will then arrange a mutually convenient time for data collection to take place. Within data collection (focus groups or one to one interviews), having checked on-line consent has already been given, and after exploring any remaining unanswered

questions raised by the Participant Information Sheet, the participants will be asked to also give recorded oral consent to participate. This consent will be recorded on the audio/video file and the researcher. Interviewees will be reimbursed for their time (vouchers).

4.2 Arm A

4.2.1 LFD testing for Covid-19 with sickness support payment

The multicomponent testing intervention will comprise a maximum of four modules that will be delivered in combination:

Module 1: Support payments for staff to enable them to self-isolate when unwell
UKHSA are providing a maximum of £2.2m to fund support payments to staff, with an estimated daily cost to providers of £60 per day for staff sickness absence. We will agree parameters on the use of staff sickness payments with providers and UKHSA (e.g. maximum payment per individual, duration of payment), but responsibility for administering payments to staff will be devolved to providers. Providers will be required to audit the use of sickness payments and provide records of how many staff have received them (and duration of payment) to the trial team and UKHSA.

Module 2: Branding and messaging around testing
Our intervention development work suggests that branding the intervention and persuasive messaging is likely to be effective in promoting engagement with testing, to consolidate the considerable existing professional expertise around testing. It is also important to revisit beliefs about LFD efficacy and ensure staff understand the importance of testing compliance within the trial. The best approach to branding and messaging will be determined in the stakeholder engagement meetings.

Module 3: Accessible training, protocols and planning
Recognising the high turnover of staff in care homes there is a need for simple protocols that unambiguously detail how to record and manage tests and test results, and for tool kits to assist with planning for staff absence contingencies. Such materials are already in place in many care homes but may not be used. We will explore whether there is value in developing such materials as part of the intervention in the stakeholder workshops.

Module 4: Regular asymptomatic staff testing for COVID-19
Care homes participating in the intervention arm of the trial will be provided with LFDs. We will consider the following two testing approaches in the intervention arm, one of which will be implemented following the intervention development work:

1. Daily pre-shift testing for staff with LFDs
2. Twice weekly asymptomatic testing with LFDs

Asymptomatic testing will be in addition to symptomatic testing for staff and residents and testing as part of the outbreak response (which we anticipate will continue to be recommended policy for all care homes for the duration of the trial).

To ensure that increased detection of asymptomatic, low-risk cases of COVID-19 does not result in unnecessary care home closures, care homes in the intervention arm with 2 or more asymptomatic cases in staff will be asked to contact the trial team rather than their local health protection team in the first instance. The trial team will undertake a risk assessment to determine whether local public health involvement is required following an initially agreed draft algorithm (Appendix A) which is being co-developed with input from representatives of UKHSA Health Protection Teams. The decision of whether to refer cases to local HPTs will be made by a suitably qualified member of the trial team (Consultant in Public Health).

4.3 Arm B

4.3.1 Usual Care

Residents and staff in non-intervention care homes will be subject to the testing policy that is in place nationally at the time of the trial. We anticipate this will be symptomatic testing, and testing as part of the public health outbreak response for residents and staff. However, if the community incidence of infection increases substantially or a new variant emerges during the trial period, it is possible that national policy will change to recommend twice weekly asymptomatic testing in staff, and hence this would also be in place in the control group.

Care home staff in control homes will not receive support payments (Module 1) and testing in control homes will not be supported by branding or messaging (Module 2). The degree to which training and testing protocols are already in place in control homes (Module 3) will be investigated in the stakeholder workshops.

Care homes in the usual care arm will be expected to follow national public health guidance for reporting suspected outbreaks to their local health protection teams. An outbreak consists of 2 or more positive (or clinically suspected) linked cases of COVID-19 that occur in the same setting within a 14-day period (this means where the cases are linked to transmission in the care setting). This applies to both staff and residents, and includes PCR and LFD results.[43] If the care home manager suspects an outbreak in the care home, they should contact the health protection team (HPT) (or community infection prevention and control team, local authority, or integrated care board (ICB) in accordance with local protocols) who will conduct a risk assessment, including whether the cases are likely to be linked. Following this risk assessment, the HPT may advise whole home outbreak testing.[43]

4.4 Interruptions and Discontinuations

Care homes may become overwhelmed and unable to implement fully the intervention if large numbers of asymptomatic staff test positive, and it is impossible to identify/employ sufficient numbers of temporary staff. In this circumstance homes will be asked to notify their provider's project manager and to continue implementing the intervention as best they can, for example by reducing the frequency of testing if necessary, and to resume the full intervention as soon as this becomes possible. At all times homes will remain in the trial unless a provider request is made formally to withdraw them, and should continue to provide trial

data as requested irrespective of whether they are able to implement the intervention in full, or indeed at all.

The trial team will produce an interim summary report after approximately three months of operating the intervention. This report will be reviewed by the IDMC, which will have the remit to recommend discontinuation of the trial. However, we do not anticipate that such a decision would be made on the basis of the primary outcome alone, as there will be a time delay in the collation of all available data on COVID-associated hospital admissions due to a lag in ICD10 coding.

4.5 Concomitant Care

Treatment for any other conditions should be given as per standard care. Participants that are eligible for VIVALDI-CT can be enrolled in any other research projects.

4.6 Blinding Considerations

It is not possible to blind the participant or the trial research teams to intervention group, therefore this will not be any attempt to conceal the allocated group

4.7 Protocol Intervention Discontinuation

Staff who are participating in the testing intervention can stop involvement in the trial at any time for any reason, and do not need to justify their reasoning if they do not wish to do so.

4.8 Compliance and Adherence

The importance of compliance with the testing intervention in care homes that are taking part in the trial will be made clear during feasibility discussions with the site's PI and other key staff working on the trial. This in no way limits the ability of any individual to opt out from the outset of the trial intervention period or discontinue participation mid-intervention.

Site adherence to the intervention will be assessed by monitoring rates of LFD testing in staff using routinely collected data that are held in the COVID-19 Datastore.[44] At the intervention development stage we will also explore whether it is feasible for each care home to prospectively record staff members participation in testing, and this will also be assessed qualitatively in the process evaluation. Sites with low adherence rates in a given month may be invited to teleconference calls with the CCTU team to discuss best practice in terms of encouraging participation at both site and individual-level.

4.8.1 Withdrawal of Consent/assent

Staff and residents have the option of opting out from the processing and analysis of their individual-level data within this study at any time during the study. Notice of the choice to opt out would be submitted to care home providers, with their details passed to NHSE, using the CAG approved process that is already in place for care homes in the VIVALDI study. The associated pseudo-identifier would then be added to an opt-out list within the Foundry system. The presence of a pseudo-identifier on this list will prevent export of any individual level data for that person to the UCL DSH, and so they will be excluded from all data processing and analysis by the research team.

If an individual opts out then they would be removed from all further data exports from Foundry and from all further analyses, including those for the final study report. However, they would still be present in any prior exports to the UCL DSH, including those for data monitoring checks and the trial's interim analysis. As such, no data exports to the DSH will be made within the first 4 weeks of the trial intervention period. However, we also note that the data exported to the trial team will be pseudonymised, and so will not constitute a direct breach of confidentiality. Further guidance can be found in the VIVALDI-CT Participant Management Plan.

4.9 Eligibility Criteria for Individuals Performing the Interventions

The trial staff will be those already working at the care home, who have been identified as qualified and available to support this research project and, where appropriate, will be supported by dedicated staff working at the care home head offices.

5 Assessments & Follow-Up

5.1 Outcomes

5.1.1 Primary Outcome

The primary outcome is the incidence of COVID-19 related hospital admissions in residents, defined as admissions with a relevant ICD-10 codes (COVID hospitalisations to be defined as any hospital admission record with a primary or secondary ICD10 code of 'U071') and/or admissions in residents who test positive for COVID-19 within 24h following admission or in the 7 days before hospital admission. This has been selected because it is the most important outcome for policymakers.

It is very difficult to differentiate hospital admissions that are caused by COVID-19 from hospital admissions where COVID-19 is an incidental finding using routine data. However, the rate of all-cause hospital admission in residents for reasons unrelated to COVID-19 is high (around 10% over a 3-month period) due to the advanced age and high levels of comorbidity in residents, which prohibits its use as the primary outcome. The incidence of all-cause unplanned (non-elective) hospital admission in residents will therefore be included as a key secondary outcome, recognising that elective admissions are extremely rare in care home residents due to their advanced age and frailty.

5.1.2 Secondary Outcomes

Although we have adopted a healthcare/NHS perspective for the primary outcome, we recognise the importance of capturing outcomes that are relevant to the social care sector, such as outbreaks and care home closures. This is reflected in our choice of secondary outcomes, which include:

- Incidence of hospital admissions (all-cause) in residents for non-elective care;

- Incidence of COVID-associated mortality* in residents;
- Incidence of all-cause mortality in residents;
- Testing uptake in staff;
- Prevalence of COVID-19 among staff and residents who test;
- Outbreaks (incidence and duration);
- Testing metrics e.g. staff time taken to conduct the test at work, costs per test;
- Impact of testing on resident, staff and visitors assessed via Social Care related Quality of Life (SCRQoL) collected via interviews in WP3B;
- Feasibility / acceptability of the testing intervention collected via interviews in WP3A;
- Care home closures due to outbreaks;
- Staff sickness absence;
- Employment of agency staff

*COVID-associated mortality will be defined as death within 28 days of a positive SARS-CoV-2 test and/or COVID-19 recorded as primary or secondary cause of death on the death certificate (using ICD-10 coding).

5.1.3 Economic Outcomes (WP4)

We will take cost-effectiveness and cost-consequences approaches, taking both an NHS and Personal Social Services (PSS), as well as a societal perspective.

We will examine the cost-effectiveness of the intervention in terms of the primary trial outcome - number of COVID-19 related hospital admissions in residents (in-trial economic evaluation).

Secondary outcomes (in-trial economic evaluation):

1. We will examine cost-effectiveness in terms of the secondary outcomes of cases prevented and resident deaths prevented.
2. We will examine the outcomes of hospital admission and number of outbreaks alongside costs offset/additional costs incurred in a cost-consequences analysis.

5.1.4 Exploratory Analyses (WP3B)

Despite the existence of well-established tools for assessing quality of life in adults, this was not well characterised during the COVID-19 pandemic and remains an important research gap. The Quality Adjusted Life Year (QALY) central to health economics cannot capture the impact of COVID-19 related measures on residents in their last years of life, for example due to reduced care due to low staffing levels, loss of communal activities, and restrictions on visitors. We are looking to fill this gap through exploratory analyses of customised data collection in a subset of care homes within the trial.

We will investigate the impact of the testing intervention, COVID-19 infection and outbreaks on social care related quality of life in residents using the Adult Social Care Outcomes Toolkit

(ASCOT), a well-established tool for measuring social care related quality of life (SCRQoL). A preference-weighted utility measure with eight conceptually distinct domains of SCRQoL, it has four “basic” domains (food and drink, accommodation cleanliness and comfort; personal cleanliness and comfort, and personal safety). These are complemented by four “higher order” domains (control over daily life; social participation and involvement; occupation, and dignity). ASCOT has been adapted for use in a range of populations including people with cognitive deficit, and by proxies such as carers.

Data for the SCRQoL analyses will be collected in WP3B. This will establish an ASCOT cohort of six care homes stratified by type and size, distributed equally across control and intervention arms in the RCT. Homes taking part in the qualitative interviews in WP3A are excluded to avoid over-burdening participating care homes.

The ASCOT tool will be used to assess SCRQoL at baseline, during/immediately after an outbreak or mid study, and at end of the study. If in-person resident interviews are not possible, Essential Caregivers (designated visitor allowed even during outbreaks) or members of staff will be interviewed using an appropriate ASCOT instrument. ASCOT data will be combined with individual level demographic and health and aggregate home level data obtained from the RCT dataset to give a comprehensive description of SCRQoL. Overall we aim to undertake approximately 30 interviews per home. Outputs will also be integrated with findings from WP3A to provide a holistic assessment of the acceptability and feasibility of the testing intervention.

5.2 Data sources

In order to facilitate set-up of the trial and to minimise the burden on care home staff, much of the data for analysis will be obtained from routinely collected healthcare information that is held within the UK COVID-19 Datastore.[44] This will include results of LFD and PCR tests for SARS-CoV-2, information on hospital admissions and deaths, and contextual information on the rollout of booster vaccinations. Data within the COVID-19 Datastore are linked to a pseudonymised ID code at the level of each individual, which can be linked to CQC IDs for participating care homes and associated staff or resident status. More information on data security and processing is given in Section 7 of this document.

Data on hospital admissions are linked to ICD10 diagnostic codes (which include specific codes for COVID-19) within the COVID-19 datastore. However, there is a lag of several months in the assignment of ICD10 codes to hospital admission data. As such, in order to allow timely monitoring of the primary outcome of the study, and avoid the risk of omitting or double-counting hospital admissions in residents, care providers will be asked to upload to the COVID Datastore weekly lists of COVID-associated hospital admissions occurring at participating care homes within the trial period. These data will also be linked to pseudonymised ID codes for each individual, allowing comparison to the routinely collected hospital admission data once available.

In order to be able to express and analyse primary and secondary outcomes as incidence rates, we require information on the denominator i.e. the total number of residents at each participating care home. This will be collected at weekly intervals in batch from each care provider. We will also collect the total number of staff and explore whether it is feasible to collect data on the number of staff who opt out of asymptomatic testing (in the intervention arm) on a weekly basis for each home.

Care home level data will be collected from providers on dates of care home closures, use of disease control measures, staff sickness absence and employment of agency staff to inform health economic analyses. We will explore the feasibility of collecting care home level data on fees paid by residents who are funded by the local authority.

Data on outbreak events (dates, size) will be obtained from the UKHSA Adult Social Care Team. Data on the local incidence of COVID-19 and co-circulation of other respiratory viruses will be obtained from the UKHSA and/or the ONS Covid Infection Survey.

Further detail on data sources is provided in section 7.

5.3 Trial Pathway

Trial visit number	Baseline	Care home data collection period		***Data source***					
**Month**	**Month 0**	**Months 1-4**		***Survey***	***Routine COVID-19 datastore***	***Uploaded to Foundry by provider data manager***	***Weekly collection from providers***	***Monthly collection from providers***	***Participants***
		Sites on Intervention arm	Sites on Standard Care arm						
Demography	X			*X*	*X*				
Resident registry	X			*X*					
Vaccination status	X				*X*				
Care home characteristics	X			*X*	*X*				
Number of residents per home (weekly)		X	X				*X*		
COVID-associated hospital admission events in residents		X	X		*X*	*X*			
Number of staff per home (weekly)		X	X				*X*		
Number of staff opting out of asymptomatic testing (monthly)*		X						*X*	
Routine data on LFD and PCR tests (staff+residents)		X	X		*X*				
Routine data on hospital admissions (residents)		X	X		*X*				
Routine data on mortality (residents)		X	X		*X*				
Outbreak event data		X	X				*X*		
Safety reporting		X	X				*X*		
**Process Evaluation (3a)**									
Focus groups		X	X						*X*
Interviews		X	X						*X*

* if the control arm starts routine testing then collected from both arms

5.4 Post-trial Care

Post-trial care will be standard medical care (routine rehabilitation).

6 Safety reporting

The principles of GCP require that both investigators and Sponsors follow specific procedures when notifying and reporting adverse events or reactions in clinical trials. These procedures are described in this section of the protocol. **Section 6.1** lists definitions, **Section 6.3** gives details of the investigator responsibilities and **Section 6.4** provides information on CCTU responsibilities.

6.1 Definitions

This is a low-risk pragmatic cluster randomised controlled trial. The safety of lateral flow testing is well established with the main risk being epistaxis.
For the purposes of this trial the only adverse events that need to be reported are ones considered a) to meet the 'seriousness' threshold, and b) to be related to the intervention.

Definitions of harm of the EU Directive 2001/20/EC Article 2 based on the principles of GCP apply to this trial.

Table 1: Definitions

<table>
<tr><td>Adverse Event (AE)</td><td>Any untoward medical occurrence in a patient or clinical trial participant using an intervention and which does not necessarily have a causal relationship with this product.</td></tr>
<tr><td></td><td></td></tr>
<tr><td>Serious Adverse Event (SAE)</td><td>An adverse event, adverse reaction, or unexpected adverse reaction that:
• results in death
• is life threatening*
• requires hospitalisation or prolongation existing hospitalisation**
• results in persistent or significant disability or incapacity
• consists of a congenital anomaly or birth defect
• is another important medical condition***</td></tr>
<tr><td colspan="2">* The term life threatening here refers to an event in which the patient is at risk of death at the time of the event; it does not refer to an event that might hypothetically cause death if it was more severe (e.g., a silent myocardial infarction)
** Hospitalisation is defined as an in-patient admission, regardless of length of stay, even if the hospitalisation is a precautionary measure for continued observation. Hospitalisation for pre-existing conditions (including elective procedures that have not worsened) do not constitute an SAE</td></tr>
</table>

*** Medical judgement should be exercised in deciding whether an AE or NAE is serious in other situations. The following should also be considered serious: important AEs or NAEs that are not immediately life threatening or do not result in death or hospitalisation but may jeopardise the participant or may require intervention to prevent one of the other outcomes listed in the definition above (e.g., a secondary malignancy, an allergic bronchospasm requiring intensive emergency treatment, seizures, or blood dyscrasias that do not result in hospitalisation, or development of drug dependency).

6.2 Adverse Events

Adverse events include:

- An exacerbation (i.e., increase in the frequency or intensity) of a pre-existing illness, episodic event or symptom (initially recorded at the baseline visit), that is detected after trial intervention
- Occurrence of a new illness, episodic event or symptom, that is detected after trial intervention

Adverse events do NOT include:

- Medical or surgical procedures: the condition that leads to the procedure is the adverse event
- Pre-existing disease or a condition present before treatment that does not worsen
- Hospitalisation where no untoward or unintended response has occurred e.g., elective cosmetic surgery
- Overdose of medication without signs or symptoms

Staff at the sites randomised to test will report the occurrence of Related AEs. Only events that are clearly **attributable to the research LFTs** (e.g. nose bleed lasting for more than 5 minutes within 30 minutes of performing the LFT) **and serious** will be considered a reportable serious adverse event, recorded in the trial database and reported to the UCL CCTU. Site personnel will complete a SAE report within 24 hours of notification of the event.

6.3 Investigator responsibilities

6.3.1 Investigator Assessment

6.3.1.1 Seriousness

When an AE occurs, the investigator responsible for the care of the participant must assess whether or not the event is 'serious' **using the definition given in Table 1** in the section above.

6.3.1.2 Causality

The investigator must assess the causality of all SAEs in relation to the trial intervention using the definitions in Table 2. There are five categories: unrelated, unlikely, possibly, probably, and definitely related.

If the causality assessment is unrelated or unlikely to be related, the event is classified as an 'unrelated SAE'. If the causality is assessed as possibly, probably or definitely related, then the event is a 'related SAE'. **Note that only 'related SAEs' should be reported for this trial.**

Table 2: Assigning Type of SAE Through Causality

Relationship	Description	Event type
Definitely	There is clear evidence to suggest a causal relationship and other possible contributing factors can be ruled out	Related SAE
Probably	There is evidence to suggest a causal relationship and the influence of other factors is unlikely	Related SAE
Possibly	There is some evidence to suggest a causal relationship (e.g., because the event occurs within a reasonable time after administration of the trial medication). However, the influence of other factors may have contributed to the event (e.g., the participant's clinical condition, other concomitant treatment)	Related SAE
Unlikely	There is little evidence to suggest that there is a causal relationship (e.g., the event did not occur within a reasonable time after administration of the trial medication). There is another reasonable explanation for the event (e.g., the participant's clinical condition, other concomitant treatment)	Unrelated SAE
Unrelated	There is no evidence of any causal relationship	Unrelated SAE

A related Serious Adverse Event is any untoward medical occurrence in a participant, which does have a causal relationship with the study procedures and is serious.

6.3.1.3 Severity or Grading of Adverse Events

The severity of all 'related' SAEs in this trial should be graded using the toxicity gradings in the Common Terminology Criteria for Adverse Events (CTCAE) Version 5.0 (2017).
Grades for SAEs according to the CTCAE are as per Table 2 below.

Table 3: Toxicity Gradings for Adverse Events

Grade	Description
One	Mild; asymptomatic or mild symptoms; clinical diagnostic observations only; intervention not indicated
Two	Moderate; minimal, local or non-invasive intervention indicated; limiting age-appropriate instrumental activities of daily living (ADL)* (e.g. nasal packing, cauterization; topical vasoconstrictors)
Three	Severe or medically significant but not life threatening; hospitalisation or prolongation of hospitalisation indicated; disabling; limiting self-care ADL**

	(Transfusion; invasive intervention indicated e.g., haemostasis of bleeding site)
Four	Life threatening consequences: urgent intervention indicated
Five	Death related to AE or AR
**Instrumental ADL refer to preparing meals, shopping for groceries or clothes, using the telephone, managing money etc.<br>** Self-care AD refer to bathing, dressing and undressing, feeding self, using the toilet, taking medications and not bedridden.*	

6.3.1.4 Expectedness

If there is at least a possible involvement of the trial intervention, **the CCTU delegated clinician** must assess the expectedness of the event. However, the Sponsor has the overall responsibility for determination of expectedness. A list of expected occurrences resulting from the trial intervention are as follows:

- Epistaxis (longer than 10-15 minutes)

When the event falls outside of this list, it must be deemed 'unexpected' by the clinical reviewer. If an SAE is assessed as being related to the trial intervention (resulting from administration of any of the research procedures) and unexpected, then the event must be reported to the REC (see Notifications sections of the protocol).

6.3.2 Notifications

6.3.2.1 Notifications by the Investigator to CCTU

CCTU must be notified of all SAEs considered to be **possibly/probably/definitely related** to the participant's involvement in the trial's intervention (LFD testing) immediately when site staff become aware of the event (in no circumstances should this notification take longer than 24 hours).

SAEs that are considered unlikely to be or unrelated to the participant's involvement on the VIVALDI-CT trial will not be recorded or reported in this study. The study is classified as low risk (the risk to participants taking part is no higher than standard care).
Investigators should use the above guidance/timelines to notify CCTU of any SAEs occurring from the time of site randomisation until 30 days after the intervention has ceased.

Related SAEs will be reported using the VIVALDI-CT Serious Adverse Event Case Report Form. This must be completed by an appropriate care home provider staff member with attention paid to the grading, causality and expectedness of the event.

The minimum criteria required for reporting an SAE are the primary event term, site name, initials and partial date of birth, name of reporting investigator and sufficient information on the event to confirm seriousness. Any further information regarding the event that is

unavailable at the time of the first report should be sent to the CCTU as soon as it becomes available.

Further details can be found in the VIVALDI-CT Safety Management Plan.

6.4 CCTU responsibilities

The Chief Investigator or a medically qualified and delegated individual will review all SAE reports received. The causality assessment given by the local investigator at the site cannot be overruled; in the case of disagreement, both opinions will be provided in any subsequent reports.

The Chief Investigator or a medically qualified and delegated individual will review the form for expectedness against the events given in Section 6.3.1.4, and provide a Sponsor assessment of expectedness for the purposes of onward reporting. The site team should not perform this function.

CCTU is undertaking the duties of trial sponsor and is responsible for the reporting of unexpected SAEs to the REC as appropriate. CCTU will keep investigators informed of any safety issues that arise during the trial.

An Annual Progress Report (APR) will be submitted to the REC within 30 days of the anniversary date on which the favourable opinion was given, and annually until the trial is declared ended.

6.4.1 Urgent Safety Measures

The CCTU or investigator may take appropriate urgent safety measures in order to protect research participants against any immediate hazard to their health or safety.

Where urgent safety measures are taken the CCTU and CIs shall immediately (not later than 3 days from the date measures are taken), give written notice to the REC of the measures taken and the circumstances giving rise to those measures, according to the relevant CCTU SOP.

7 Quality Assurance & Control

7.1 Risk Assessment

The Quality Assurance (QA) and Quality Control (QC) considerations for the VIVALDI-CT trial are based on the standard CCTU Quality Management Policy that includes a formal Risk Assessment, and that acknowledges the risks associated with the conduct of the trial and proposals of how to mitigate them through appropriate QA and QC processes. Risks are defined in terms of their impact on the rights and safety of participants; project concept including trial design, reliability of results and institutional risk; project management; risk benefit of the trial; and other considerations.

QA is defined as all the planned and systematic actions established to ensure the trial is performed and data generated, documented and/or recorded and reported in compliance with the principles of GCP and applicable regulatory requirements. QC includes the operational techniques and activities performed within the QA system to verify that the requirements for quality of the trial related activities are fulfilled.

The VIVALDI-CT Risk Assessment has been reviewed by the CCTU's Quality Management Group (QMG).

7.2 Central Monitoring at CCTU

CCTU staff will review data and other information provided by investigators to identify trends, outliers, anomalies, protocol deviations and inconsistencies. The frequency and type of central monitoring will be detailed in the VIVALDI-CT Quality Management and Monitoring Plan (QMMP).

7.3 Monitoring

The frequency, type and intensity of routine on-site monitoring and the requirements for triggered on-site monitoring will be detailed in the VIVALDI-CT QMMP, including any provision for remote or self-monitoring. The QMMP will detail the procedures for review and sign-off of monitoring reports. In the event of a request for a trial site inspection by any regulatory authority UCL CCTU must be notified as soon as possible.

Essential trial issues, events and outputs, including defined key data points, will be detailed in the VIVALDI-CT Data Management Plan.

7.3.1 Direct access to Participant Records

Participating investigators must agree to allow trial-related monitoring, including audits, EC review and care home data, by providing access to source data and other documents as required.

7.3.2 Confidentiality

All data will be handled in accordance with the Data Protection Act 2018, the UK General Data Protection Regulation (UK GDPR) and subsequent updates and amendments.

As previously described, informed consent for testing will not be sought.

Confidentiality will be maintained as follows:

1. Routinely collected, pseudonymised data on uptake and outcomes of COVID-19 testing are held in the COVID-19 datastore. These data will be transferred securely to the UCL data safe haven for analysis by the UCL research team. The Data Safe Haven[45] has been certified to the ISO27001 information security standard and conforms to NHS Digital's Information Governance Toolkit. Built using a walled garden approach, where the data is stored, processed and managed within the security of the system, avoiding the complexity of assured end point encryption. A file transfer

mechanism enables information to be transferred into the walled garden simply and securely.

2. Identifiable data (NHS number, name, DOB, sex) on residents who are admitted to hospital during the testing period will be collected by each participating care home and sent securely to their organisation's data manager. The data manager will collate lists of identifiable data and send them securely to NHS England who will pseudonymise and link the datasets to data on hospital admissions before making them available to the research team.
3. For data collection undertaken in work package 1 and study 3a, the signed informed consent forms will carry participants name and appropriate simple electronic signature). These will be stored securely at University of Strathclyde. These consent forms will only be accessed by UCL CCTU staff for purposes of monitoring the consent procedure.
4. Aggregate, non-identifiable data that are collected by each care home as part of the trial (e.g. use of disease control measures recorded weekly, number of staff opting out of testing) will be collated by each providers' data manager and transferred securely to the UCL Data Safe Haven. Original copies of the trial CRFs will be stored locally by each care home.

7.4 Source Data

For this trial, the majority of datapoints (on primary outcome along with routinely collected data) will be recorded directly into the UK COVID Datastore and therefore this dataset will be regarded as source data. Additional aggregated weekly data will be collected for each care home from care providers, with the raw data provided considered source data for the trial.

Limited aggregate data on the use of disease control measures and administrative records (e.g. dates of care home closures, costs of temporary staff) will be obtained directly from care homes. Interviews undertaken as part of the process evaluation and exploratory analyses will be audio-recorded. The location of each datapoint will be detailed in the VIVALDI-CT Data Management Plan.

A Source Data Agreement will be put in place as part of the site activation process with each site. This will define the source documents and the data therein, together with location of the source documents.

All trial data should be verifiable from source documents, which may include CRFs/eCRFs, paper notes and electronic health records.

7.5 Data Collection and Transfer Methods

Identifiable data will be transferred from providers' designated data manager to NHSE using secure file transfer protocols (FTP).

Data processing, merging and analysis scripts will be structured, and iterations of the dataset for analysis will be labelled and stored within the UCL DSH following CCTU SOPs.

The data on the UCL DSH will only be accessible to members of the VIVALDI-CT trial team at CCTU, delegated site staff and external regulators if requested.

7.6 Data Management

The Trial team will regularly export specifically and routinely collected data from the COVID datastore to the UCL DSH, and will monitor these data for quality and completeness. Analysis scripts will be structured, and iterations of the dataset for analysis will be labelled and stored within the UCL DSH following CCTU SOPs.

For the weekly aggregate data collected from the care providers for each site, data collection, data entry, queries raised by a member of the VIVALDI-CT trial team and database lock(s) will be conducted in line with the CCTU SOPs and VIVALDI-CT Data Management Plan. After completion of the trial, the database will be retained for on-going analysis of secondary outcomes.

The database will be password protected and only accessible to members of the VIVALDI-CT trial team at CCTU, delegated site staff and external regulators if requested. Database users will only be granted permissions to use the database functionality appropriate to their role in the clinical trial.

7.7 Data Storage

Individual-level trial data will be stored in the UCL Data Safe Haven[45] which is hosted by UCL. The Data Safe Haven has been certified to the ISO27001 information security standard and conforms to NHS Digital's Information Governance Toolkit. Built using a walled garden approach, where the data is stored, processed and managed within the security of the system, avoiding the complexity of assured end point encryption. A file transfer mechanism enables information to be transferred into the walled garden simply and securely.

All PII will be held only by individual care homes or NHSE. Consent forms obtained via interviews and focus groups will be scanned and stored in the secure trial databases. These databases are protected by multi-layer firewalls with full data encryption at rest and in transit.

For interviews, upon completion of transcription the pseudo-anonymised data will be stored on both the Strathclyde OneDrive network in a secure folder – with additional copies being held at UCL within the UCL Data Safe Haven. Only the named investigators having access. All names & identifying information will be removed from the transcripts, and contact details & consent forms reflecting the participants will be held in a secure file elsewhere by the research team. Raw data will be destroyed after transcription & checks have been carried out.

7.8 Data Archiving

Once all primary, secondary and health economic and exploratory analyses have been completed the trial data will be archived. Once the trial data has been archived the trial database will be decommissioned and will no longer be available. Any subsequent/ further analysis will be performed using the archived data.

The site investigators agree to archive and/or arrange for secure storage of VIVALDI-CT trial materials and records/essential documents for a minimum of 5 years after the close of the trial unless otherwise advised by the CCTU.

7.9 Quality Issues

Quality Issues are issues that can have an impact on patient safety, rights, and well-being; data integrity and/or scientific rigor; and compliance with regulatory requirements; these can be classified as protocol deviations, potential serious breaches, near misses etc.

A protocol deviation is any departure from procedures documented in this protocol, this includes deviations that cannot be predicted. If a protocol deviation is identified the VIVALDI-CT trial team should be contacted and CCTU's protocol deviation reporting process will be followed.

A 'serious breach' is a deviation from procedures documented in this protocol, GCP or other clinical trial regulations that is likely to affect to a significant degree:

1. The safety or physical or mental integrity of the participants in the trial, or
2. The scientific value of the trial

If a serious breach is suspected the CCTU must be contacted within 1 working day. The CCTU and CI will prepare a report of the serious breach, which will be reviewed by the Sponsor. If appropriate, the Sponsor will report it to the REC within 7 days.

8 Statistical Considerations

8.1 Sample Size

Based on recent Vivaldi data we found that over a 3-month period 1.8% of residents had a COVID-19 related hospital admission, and the Intra-cluster correlation (ICC) across homes was 0.003 (95% CI 0.000-0.007). As the trial collects data over 5-6 months until final analysis we assume a cumulative incidence of around 3.0% in the routine care arm and conservatively an ICC of up to 0.01 (higher in line with the higher cumulative incidence compared to 3 months), an average care home size of 35 residents with coefficient of variation in size of 0.5. With a total of 280 homes randomised 1:1 to trial arms and taking the usual two-sided testing at 5% significance level the design provides 84% power to detect a reduction in COVID-19 related admissions due to intervention to 1.9% (relative risk 0.63).

8.2 Assignment of Intervention

8.2.1 Randomisation

Care homes will be randomised to standard care (testing policy that is in place at the time of trial) versus intervention (multi-component testing intervention) in a 1:1 ratio. If all providers are ready for trial participation at the same time then all participating homes will be randomised at the same time. Otherwise the homes from different providers will be randomised in a phased approach, as they become ready.

8.2.2 Sequence generation

Randomisation will be performed by the trial statistician after enrolment of care homes and prior to implementation of the intervention, based on pseudo-random number generation. Restricted randomisation (specifically covariate constrained randomisation) will be used to ensure balance on care home provider, size and region.

8.2.3 Blinding

The proposed trial will be open (non-blinded). It is not possible to blind employees or researchers to the intervention allocation.

8.3 Statistical Methods

8.3.1 Statistical Analysis Plan

A detailed statistical analysis plan (SAP), including a full specification of the analysis principles and details, will be drafted as early as possible and finalised prior to the first substantive analysis, following approval by the Program Steering Committee (PSC) and review by the Data Monitoring and Ethics Committee (DMEC).

8.3.2 Interim Analyses

A single interim report will be produced after approximately 3 months of operating the intervention to allow an independent committee to assess the emerging benefit / risk ratio, and process outcomes such as the actual level of testing in both trial arms. It may not be possible to report on all study outcomes at this point, and so analysis will focus on the COVID-associated hospital admissions data collected specifically from care home providers for the trial, along with evaluation of staff testing activity in the trial arms and any issues that have been raised during feedback from sites. The committee will recommend trial termination in the unlikely event of substantial negative effects, or no clear difference in testing activity between arms.

In the event that a significant benefit (reduction in COVID-associated hospital admissions) is demonstrated (due to higher than anticipated rates of COVID-19 between November 2022 and February 2023) at interim analysis the trial will nevertheless continue to planned completion (i.e. 6 months duration) to deliver more precise data for cost-effectiveness analysis and to allow for a full evaluation also incorporating routinely collected coded hospital admission data. The trial will however stop if at any time the routine care testing policy across homes changes to closely resemble the intervention strategy (e.g. by including sick-pay alongside asymptomatic testing). The committee may also make a recommendation to extend the total trial duration should hospital admission incidence be lower than anticipated, or variability between homes be higher than anticipated.

8.3.3 Statistical Methods – Overview

Analysis of the primary outcome, and secondary outcomes expressed as event incidence, will be based on Poisson or negative binomial regression with cluster-robust standard errors, adjusting for calendar time and key care home characteristics used in the restricted

randomisation such as provider, region and size. Unadjusted effect estimates from these analyses will be reported for completeness. All analyses will be pre-specified in a Statistical Analysis Plan.

8.3.4 Statistical Methods – Coding of outcomes

Events defining the primary outcome of incidence of COVID-associated hospital admission will be recorded specifically by providers for this trial, and may also be derived from routinely collected SARS-CoV-2 testing and hospital admissions data. For the routinely collected hospital admissions data, there is a lag of several months before ICD-10 coding becomes available. Early analysis will therefore be based on the specially collected trial data alone, but data quality will be evaluated with reference to the routinely collected data. A decision on the final coding for the primary outcome using these different data sources will be made when the SAP is drafted prior to the final statistical analysis being conducted.

8.3.5 Additional Analyses - Subgroup

Using interaction terms, we will explore whether the effect of the intervention on the primary outcome differed between time periods defined by the national recommendations for testing in the routine care arm, should these change. To allow better consideration of the likely intervention impact across the whole care home sector we will also explore whether the intervention effect differs according to care home size, and other characteristics such as proportion of temporary staff.

8.3.6 Additional Analyses - Adjusted

Adjusted analyses are primary.

8.3.7 Analysis Population and Missing Data

Our primary analysis for the primary outcome will include all trial care homes (intention to treat). We will however also define an implementation score based on the frequency and proportion of staff testing at each home based on data the homes provide, which may vary over time. In exploratory analysis we will assess whether the primary outcome is associated with this implementation score within the intervention arm, and express the effect of the intervention relative to control arm for different levels of implementation. This analysis will be based on the same regression method as used for the primary analysis.

9 Economic Evaluations

9.1 Economic Analysis

The health economic analysis of VIVALDI-CT will investigate the cost-effectiveness and cost-consequences of the testing intervention taking both a NHS, Personal Social Services, and a societal perspective. We will also explore the short and long-term costs of the testing intervention under different epidemiological scenarios (e.g. high/low community incidence of infection).

Aim: To estimate the cost-effectiveness and cost-consequences of the testing intervention

We will examine within-trial costs and outcomes in intervention and control groups from each perspective. We will examine the cost-effectiveness of the intervention in terms of the primary outcome and in terms of all-cause mortality. The costs of admission will be excluded from the total costs under consideration in this case. We will also examine cost-effectiveness in terms of the secondary outcomes of cases prevented and resident deaths prevented. We will examine the outcomes of hospital admission and number of outbreaks alongside costs offset/additional costs incurred in a cost-consequences analysis.

Generalised linear models appropriate to counts (hospital admissions, numbers of outbreaks, cases) or binary outcomes (deaths) and costs will be applied. For the purposes of the cost-effectiveness analyses, these will take into account possible correlations between costs and outcomes either by non-parametric bootstrapping of separate regressions or joint modelling approaches such as seemingly unrelated regressions. Where individual level data are available, analyses will take a multilevel approach to adjust for clustering at the care home level either by two-stage bootstrapping of separate regressions or simultaneous modelling.[46],[47] Incremental cost-effectiveness ratios will be presented, net benefit calculated over a range of willingness to pay values for gains in outcomes to generate cost-effectiveness acceptability curves.

We will then build a compartmental model to study transmission of infection and infer the proportion of COVID-19 infections and deaths averted by the intervention under different epidemiological scenarios (e.g. high/low community incidence of infection). The model will consider two populations i.e. home residents and staff, and take into account both symptomatic and asymptomatic cases, as well as hospital admissions and deaths. The model will be calibrated to trial results and modelling results will be projected in time by extending the time horizon. Unit costs calculated in the first part of the economic analysis will be discounted to future years values and associated to modelling results to estimate the projected cost-effectiveness of the intervention.

9.2 Within-trial analysis

9.2.1 Outcomes

9.2.1.1 Resource Use

NHS and Personal Social Services (PSS) service use

- NHS: As the trial is not collecting data directly from participants, data collected on resident's health care use (from COVID-19 datastore) will be limited to:
  - inpatient hospital episodes
  - A&E attendance
  - antiviral treatment
- PSS: care home days of council-funded residents, self-payers

In addition we will investigate collection of other service use:

- whether it is feasible to link to other datasets that are held on healthcare usage in the COVID-19 Datastore such as primary care consultations
- management of outbreaks (see sections 5.2 and 9.2.2) including care home closures, staff sickness absence and use of agency staff

Lost production (see 9.2.2):

- work time lost to self-isolation and illness related to COVID (see section 5.2)
- care home days of self-payer residents (section 5.2)

9.2.1.2 Health related Quality of life

Health-related quality of life measures are not being collected from residents.

9.2.2 Cost Data

Methodology: We will take cost-effectiveness and cost-consequences approaches, taking both an NHS + Personal Social Services (PSS), and a societal perspective.

NHS+PSS costs: we will calculate the following costs of care for residents: secondary care costs for residents (hospital admissions, A&E, antivirals) and care home costs for council-supported residents. As the trial is not collecting data directly from participants, data collected on resident's health care use will be limited to hospitalisation (including A&E attendance) and antiviral treatment. We will investigate whether it is feasible to link to other datasets that are held on healthcare usage in NHS Foundry such as contacts with primary care. We will also investigate whether NHS and PSS costs associated with management of outbreaks can be estimated based on the available data on outbreaks. Care home costs to the NHS and to Councils with Adult Social Services Responsibilities (CASSRs) will be estimated from care home providers' submissions.

Societal costs: Lost production: work time lost to self-isolation and illness related to COVID can be considered a cost from the societal perspective. We will use a human capital approach to estimate the costs of absenteeism. Care home fees for self-payers.

9.2.2.1 Cost of the VIVALDI-CT intervention

Direct costs of the intervention: LFD costs, time spent testing at work and wage compensation paid by UKHSA and based on unit costs provided by the DHSC. We will also consider a broader set of intervention-related costs: changes to shift patterns, overtime payments to staff willing to cover shifts of staff self-isolating, payments to self-isolating staff above SSP level, filling missing shifts with agency workers (derived from routine data). Qualitative information on these issues will also be collected in the process evaluation.

9.2.2.2 Cost of Healthcare resource use

NHS and PSS service use data (derived from data linkage in the NHS Foundry) will be costed using the NHS National Cost Collection[48,49], PSSRU Unit Costs and other published sources, alongside unit costs calculated within the trial.

9.2.3 QALYs
No health-related quality of life measures are being collected directly from residents. The in-trial analysis will not make use of QALY data.

9.2.4 Analysis results

9.2.4.1 Primary analysis
Within-trial costs and outcomes in intervention and control groups from each perspective are examined. We will examine the cost-effectiveness of the intervention in terms of the primary outcome. The costs of admission will be excluded from total costs in this case.

9.2.4.2 Missing data
Missing data will be dealt with as in the main statistical analysis, with the primary analysis being an intention to treat analysis and secondary analyses taking into account assumptions about missingness patterns and multiple imputation.

9.2.4.3 Sensitivity analysis
No specific sensitivity analyses have been pre-planned within the in-trial analysis; however should investigators become aware of areas of uncertainty in the cost or outcome parameters during the trial, we will conduct sensitivity cost-effectiveness analyses to explore the impacts of uncertainty on results.

9.2.4.4 Secondary analyses
We will also examine cost-effectiveness in terms of secondary outcomes of cases prevented and resident deaths prevented. We will examine outcomes of hospital admission and number of outbreaks alongside costs offset/additional costs incurred in a cost-consequences analysis.

9.3 Modelling analysis
We will build a compartmental model of COVID-19 transmission in a care home setting. The model will account for two populations: care home staff and care home residents, and study infection transmission among them. We will model two scenarios: (i) standard care: residents and staff get tested only if they show symptoms; and (ii) intervention: standard care plus regular testing of all staff for COVID-19 as defined in work package 1.

Scenario analysis on COVID-19 prevalence in both populations and intervention scale up will allow us to obtain a range of epidemiological results that will also be projected to study the long-term impact of the intervention. Following this, costs as estimated in WP 4A will be aggregated to provide information on the affordability and budgetary impact of the proposed intervention. These costs will comprise: (i) cost of single COVID-19 case identified, (ii) incremental cost of single true positive COVID-19 case identified when using the intervention compared to the standard care. The primary outcome measure of effectiveness will be the proportion of COVID-19 cases averted and deaths averted. Results will be presented in the form of incremental cost-effectiveness ratios (ICERs) and/or incremental net benefit (INB), as appropriate. Discounting will be applied to future health benefits and costs if appropriate and

will be varied in sensitivity analysis. Results will be presented in standard formats such as projection curves, cost-effectiveness planes or cost-effectiveness acceptability curves (CEACs), etc. Univariate and probabilistic sensitivity analysis will be performed as appropriate taking into account uncertainty on costs, with results presented in for example, tornado plots. Sensitivity and scenario analysis using the model will lead to the identification of cost-effectiveness thresholds and willingness to pay thresholds.

10 Regulatory & Ethical Issues

10.1 Compliance

10.1.1 Regulatory Compliance

The trial will be conducted in compliance with the approved protocol, the Declaration of Helsinki 1996, the principles of GCP as laid down by the ICH topic E6 (R2), General Data Protection Regulation and the UK Data Protection Act 2018 and the UK Policy Framework for Health and Social Care Research.

10.1.2 Site Compliance

Agreements that include detailed roles and responsibilities will be in place between participating sites, CCTU and the Sponsor.

Participating sites will inform CCTU as soon as they are aware of a possible serious breach of compliance, so that CCTU can fulfil its requirement to report the breach to the Sponsor (see section 7.9).

10.1.3 Data Collection & Retention

Clinical notes and administrative documentation should be kept in a secure location (for example, locked filing cabinets in a room with restricted access) During this period, all data should be accessible, with suitable notice, to the competent authorities, the Sponsor, and other relevant parties in accordance with the applicable regulations. The data may be subject to an audit by the competent authorities. Medical files of trial participants should be retained in accordance with the maximum period of time permitted by the hospital, institution or private practice.

Also see section 7.8 on data archiving requirements.

10.2 Ethical Approvals

10.2.1 Ethical Considerations

The following ethical considerations, problems and/or dilemmas relating to the trial should be considered:

- Asymptomatic testing may disadvantage intervention homes if it leads to significant increases in staff sickness absence or care home closures. This may disincentivise care homes from participating.

- Policy changes affecting the control group during the trial may undermine our ability to evaluate the impact of the intervention (e.g. if asymptomatic testing is introduced as national policy during the testing period of the trial)
- If the incidence of COVID-19 is very low we will not be able to evaluate the effectiveness of the intervention
- It is uncertain whether the Government would fund staff sickness payments in the long-term even if the trial suggests they are key to the success of the intervention

10.2.2 Ethics Committee Approval

Prior to the commencement of the trial, REC approval and Health Research Authority (in England) approvals will be gained. Any substantial amendments will also be submitted and approved by the main REC and HRA. No trial sites will be located outside of England. CAG approval is also required to enable collection of identifiable data from residents without informed consent.

Before initiation of the trial at each site, the local information pack will be provided by UCL CCTU. The local information pack will contain the protocol and essential documentation for the execution of the trial. An appropriate contract with the care home provider will also be put into place.

10.3 Competent Authority Approvals

This is not a Clinical Trial of an Investigational Medicinal Product (IMP) as defined by the EU Directive 2001/20/EC. In addition, the UK Competent Authority, the MHRA, does not consider the VIVALDI-CT's use of LFDs as a medical device within their scope. Therefore, a Clinical Trial Authorisation (CTA) is not required in the UK.

The progress of the trial and safety issues will be reported via the Annual Progress Report form to the REC in accordance with HRA's requirements.

Safety reports, specifically expedited reporting for 'related and unexpected' SAEs, will be submitted to REC in accordance with the HRA's requirements.

10.4 Other Approvals

The protocol will be submitted by those delegated to do so to the relevant lead of each participating site for confirmation of capacity and capability prior to initiating trial activities at that site.

Amendments to the trial protocol will likewise be submitted for capacity and capability to each participating site prior to the amended protocol being brought into effect.

Note that site approvals may be gathered via head offices of care homes, who will liaise with individual sites and provide approvals in this manner. This does not negate the requirement for the individual care home to confirm their capacity and capability, however.

10.5 Trial Closure

Trial closure is defined as the date when all data have been received and cleaned (all data queries resolved at all sites) and the database locked for final analysis. For VIVALDI-CT this will be after all trial data received and all queries have been resolved.

The REC/HRA will be notified within 90 days of trial completion. Within one year of the end of the trial, the CCTU will submit a final trial report with the results of the trial, including any publications/abstracts of the trial, to the HRA. In the case that trial is ended prematurely, the CCTU will notify the HRA within 15 days, including the reasons for the premature termination. All reports will be reviewed and approved by the CI and Sponsor.

At a site level, a care home may only be deemed 'closed' once all trial-related activities have been reconciled and/or complete, all outstanding data queries resolved; all necessary trial documentation has been provided to the participating sites for inclusion in the ISF; following the Trial Manager or delegate at the CCTU performing a close-out visit or teleconference, as appropriate. The close-out process will be documented accordingly. Any outstanding actions resulting from the close-out visit will need to be completed and documented within an agreed timeframe between the participating site and CCTU. The responsibility for completing any outstanding actions will lie both with the local research staff and Trial Manager or delegate. A letter confirming that close out is complete has been sent to the site PI from the CCTU.

11 Indemnity

The Sponsor holds insurance to cover participants for injury caused by their participation in the clinical trial. Participants may be able to claim compensation if they can prove that UCL or partner institutions on the VIVALDI-CT trial have been negligent. However, as this clinical trial is being carried out in care home settings, the care home continues to have a duty of care to the participant in the clinical trial. UCL does not accept liability for any breach in the care home's duty of care, or any negligence on the part of any care home employees. This does not affect the participant's right to seek compensation via the non-negligence route.

Participants may also be able to claim compensation for injury caused by participation in this clinical trial without the need to prove negligence on the part of UCL or another party. Participants who sustain injury and wish to make a claim for compensation should do so in writing in the first instance to the Chief Investigator, who will pass the claim to UCL's insurers.

Care homes selected to participate in this clinical trial shall provide clinical negligence insurance cover for harm caused by their employees and a copy of the relevant insurance policy or summary shall be provided to UCL, upon request.

12 Finance

VIVALDI-CT is fully funded by an NIHR Health and Social Care Delivery Research (HSDR) Programme number [enter number]. Costs of LFDs and support payments for care home staff who are required to self-isolate will be provided by the UKHSA.

13 Oversight & Trial Committees

Trial oversight is intended to preserve the integrity of the trial by independently verifying a variety of processes and prompting corrective action where necessary.

There are a number of committees involved with the oversight of the trial. These committees are detailed below.

13.1 Trial Management Group

A Trial Management Group (TMG) will be formed comprising the Chief Investigator, other lead investigators (clinical and non-clinical) and CCTU staff and PPI contributors. The TMG will be responsible for the design, coordination and strategic management of the trial. The membership, frequency of meetings, activity (including trial conduct and data review) and authority will be covered in the TMG Terms of Reference.

13.2 Programme Steering Committee

The Trial Steering Committee (TSC) is the independent group responsible for oversight of the Programme, including the RCT, and to safeguard the interests of trial participants. The TSC provides advice to the CI, CCTU, the funder on all aspects of the trial through its independent Chair. The membership, frequency of meetings, activity (including trial conduct and data review) and authority will be covered in the TSC terms of reference.

13.3 Data Monitoring and Ethics Committee

The Data Monitoring and Ethics Committee (DMEC) is the only oversight body that has access to the confidential, accumulating data for the trial. The DMEC is responsible for safeguarding the interests of trial participants, as well as monitoring the accumulating data and making recommendations to the TSC on whether the trial should continue as planned. The membership, frequency of meetings, activity and authority will be covered in the DMEC Terms of Reference. The DMEC will consider data in accordance with the statistical analysis plan and will advise the TSC through its Chair.

13.4 Trial Sponsor

The role of the sponsor is to take on responsibility for securing the arrangements to initiate, manage and finance the trial. UCL is the trial sponsor and has delegated the duties as sponsor to CCTU via an amendment to the VIVALDI-CT collaborator agreement.

14 Patient & Public Involvement

Patient and Public Involvement (PPI) in research is defined by the Health Research Authority (HRA) as research being carried out 'with' or 'by' members of the public rather than 'to', 'about' or 'for' them.[50] The 'public' can include patients, potential patients, carers and other users of health and social care services, as well as people from organisations that represent people who use services. In some cases, this may include involvement of a trial's participants in guidance or oversight of a trial.

14.1 Potential Impact of PPI

The team recognise that effective PPI will be critical to the success of the project. In particular, it is essential that the views of families, residents and the public inform decisions about the benefits and harms of regular testing.

Patient public involvement has already informed the development of this programme, by highlighting the barriers to testing and the need to capture its adverse impacts on staff, residents and providers (WP3). Public advisors have also emphasised the importance of developing a strong plan for implementation, recognising the financial implications of long-term use of testing and sickness payments, informing our emphasis on implementation in WP5. Our lay summary was developed with colleagues from Four Seasons Healthcare and has been reviewed and improved by one carer and Liz Jones, the policy lead for the National Care Forum.

PPI activities will be overseen by Natasha Southall, who is the care quality lead for Four Seasons Healthcare. The PPI group will meet three times during the study, and 2 members of the PPI team will also participate in stakeholder workshops to ensure communication between these two groups.

The PPI team will deliver the following objectives:

- To ensure that the 'voice and views' of the public regarding regular testing for COVID-19 are heard by the research team and the wider stakeholder group
- To create an open, inclusive culture enabling effective communication between the study team, PPI group and the wider stakeholder and oversight groups
- To agree an approach to communicate outputs from the trial to different audiences, including care home staff, residents and their families and the public using a variety of media (see WP5).

14.2 Identifying PPI Contributors

The PPI lead will work with the research team to recruit a diverse (gender, age, ethnicity) PPI group (6-8 individuals) including relatives, members of the public and residents (if possible), drawing on 1) Vivaldi's existing PPI group of relatives, members of the public, and care home staff, 2) the Kent Surrey Sussex NIHR Applied Research Collaboration PPIE networks via collaborator Becky Sharp (Social Care Implementation Lead, and 3) PPIE networks in Greater Manchester via Co-I's Verma and Regan, who work with the social care inclusive research group and Vocal (https://www.wearevocal.org/) led by Prof Bella Starling, including Black Asian and Minority Ethnic (BAME) Research Advisory Group (BRAG) as part of the Greater Manchester Health Inequalities Steering Group.

15 Publication & Dissemination of Results

15.1 Publication Policy

15.1.1 Trial Results

The results of the trial will be disseminated regardless of the direction of effect. The publication of the results will comply with the UCL CCTU Publication Policy and the VIVALDI-CT specific publication policy and will include submission to open access journals.

A lay summary of the results will also be produced to disseminate the results to those participants who took part, as well as those who express an interest in the findings.

A summary of results will be included online in the publicly accessible HRA website within 12 months of date of trial closure.

15.1.2 Authorship

The TSC will advise on the nature of publications. All publications shall include a list of investigators, and if there are named authors, these should include the Chief Investigator(s), and appropriate Co-Investigators, Trial Manager, and Statistician(s) involved in the trial and/or data analysis. Named authors will be agreed by the Chief Investigator(s) and in line with the VIVALDI-CT publication policy. If there are no named authors, then a writing committee will be identified.

15.1.3 Reproducible Research

The VIVALDI-CT Trial Protocol and Statistical Analysis Plan will be published for public access.

16 Data Sharing

Data will be shared according based on the following principles:

- No data should be released that would compromise an ongoing trial or study.
- There must be a strong scientific or other legitimate rationale for the data to be used for the requested purpose.
- Investigators who have invested time and effort into developing a trial or study should have a period of exclusivity in which to pursue their aims with the data before key trial data are made available to other researchers.
- The funder requirements for data sharing need to be reflected in this section.
- The resources required to process requests should not be under-estimated, particularly successful requests which lead to preparing data for release. Therefore, adequate resources must be available in order to comply in a timely manner or at all, and the scientific aims of the study must justify the use of such resources.
- Data exchange complies with Information Governance and Data Security Policies.

Data will be available for sharing between the trial researchers as detailed in the Data Sharing Agreement between the institutions hosting the VIVALDI-CT researchers.

Requests for access to trial data will be considered, and approved in writing where appropriate, after formal application to the TSC. Considerations for approving access are documented in the TSC Terms of Reference.

17 Process Evaluation (WP3A)

17.1 Background

There is major diversity across care homes for example in terms of provision of care, resident population, care home size, and the care home workforce. As a result it is essential to consider the feasibility and sustainability of the intervention and how contextual factors might impact on the ability to scale it if the trial suggests it is effective and cost-effective. These issues will be addressed in the process evaluation.

17.2 Aims

To understand intervention roll out and identify areas for optimisation to inform future intervention scale-up, should the testing approach prove effective and cost effective

17.3 Objectives

- To determine intervention acceptability
- To determine the role of context play in shaping the way the intervention operated
- To determine what can be learned about intervention fidelity and adaptation
- To determine which intervention components worked as anticipated and which need further modification
- To investigate unanticipated intervention effects
- To determine what can be learned from the control group

17.4 Process Evaluation Outputs

- Implementation guidance and training packages ready for future scale up;
- Details of minimal care home requirements and staff competencies necessary for intervention delivery.

17.5 Study Design

Drawing on MRC guidance,[43,44] we will use the intervention programme theory and associated logic models from WP1 to undertake a parallel mixed methods process evaluation.

17.6 Theoretical framework

We will draw upon diverse theories of change and ensure we are engaging with change processes wholistically; at level of the individual (e.g., the theoretical domains framework), at the level of the team and care home (e.g., normalisation process theory[53,54]) and through understanding change mechanisms through systems science.[55]

17.7 Sample identification and recruitment

Qualitative data will be collected from 28 (10%) care homes evenly distributed across each intervention and control arms and spaced across time. Selection criteria will focus on

geography, socio-economic status of area, highest/lowest rates of infection. Within homes attempts will be made to recruit heterogeneous samples of staff. We will work closely with site PIs to ensure a range of staff are offered the opportunity to take part in the on-line focus groups or one to one interviews. We will monitor any biases in recruitment.

We will also recruit a further six focus groups with residents (with capacity to consent), relatives and visitors. These will be evenly distributed across each intervention and control arms and take place towards the end of the trial period.

17.8 Overview of Data Collection

Consent will be attained for collecting qualitative data using focus groups, and semi-structured one-to-one interviews with staff, families and visitors. For all brief sociodemographic data is collected to allow sample description. For the qualitative data combination of deductive and inductive thematic analysis will be used.[45] Quantitative data will include descriptive statistics from across all trial sites complemented by multivariate analyses of data sets where appropriate. Relevant analyses will be combined and integrated to deliver holistic answers to each of the research questions. [46–48]

17.9 Qualitative Data Analysis

Interview/focus group transcriptions and qualitative survey data will be imported into NVivo10 software to facilitate data handling, organisation and coding. Analysis will firstly be thematic and use a combination of deductive and inductive thematic analysis.[45] These data driven themes will then be further explored in relation to the TDF[60] and NPT.[61]

17.10 Reporting and Dissemination

Publication of the findings of the process evaluation will be aimed at diverse journals including those with generic readership (implementation science) as well as for specialist audiences (British Journal of Health Psychology). We will work closely with PPI to develop other outputs for other audiences.

18 Protocol Amendments

Table 4. Summary of Protocol Amendments

Protocol version	Protocol date	Summary of changes
V1.0	21 Oct. 22	Initial version

References

1. Chan AW, Tetzlaff JM, Altman DG, et al. SPIRIT 2013 statement: defining standard protocol items for clinical trials. *Ann Intern Med*. 2013;158(3):200-207. doi:10.7326/0003-4819-158-3-201302050-00583

2. Chan AW, Tetzlaff JM, Gotzsche PC, et al. SPIRIT 2013 explanation and elaboration: guidance for protocols of clinical trials. *BMJ*. 2013;346(jan08 15):e7586-e7586. doi:10.1136/bmj.e7586

3. Murray L, Convery L. The *Care Standards Act 2000*. *Hous Care Support*. 2000;3(4):29-32. doi:10.1108/14608790200000033

4. Gordon AL, Franklin M, Bradshaw L, Logan P, Elliott R, Gladman JRF. Health status of UK care home residents: a cohort study. *Age Ageing*. 2014;43(1):97-103. doi:10.1093/ageing/aft077

5. Kinley J, Hockley J, Stone L, et al. The provision of care for residents dying in UK nursing care homes. *Age Ageing*. 2014;43(3):375-379. doi:10.1093/ageing/aft158

6. Alzheimer's Society. *Dementia UK Update*.; 2014. Accessed November 27, 2021. https://www.alzheimers.org.uk/sites/default/files/migrate/downloads/dementia_uk_update.pdf

7. Levin AT, Jylhävä J, Religa D, Shallcross L. COVID-19 prevalence and mortality in longer-term care facilities. *Eur J Epidemiol*. 2022;37(3):227-234. doi:10.1007/s10654-022-00861-w

8. Kaelen S, van den Boogaard W, Pellecchia U, et al. How to bring residents' psychosocial well-being to the heart of the fight against Covid-19 in Belgian nursing homes—A qualitative study. Kotozaki Y, ed. *PLOS ONE*. 2021;16(3):e0249098. doi:10.1371/journal.pone.0249098

9. Stratil JM, Biallas RL, Burns J, et al. Non-pharmacological measures implemented in the setting of long-term care facilities to prevent SARS-CoV-2 infections and their consequences: a rapid review. Cochrane Public Health Group, ed. *Cochrane Database Syst Rev*. 2021;2021(9). doi:10.1002/14651858.CD015085.pub2

10. Telford CT, Onwubiko U, Holland DP, et al. Preventing COVID-19 Outbreaks in Long-Term Care Facilities Through Preemptive Testing of Residents and Staff Members — Fulton County, Georgia, March–May 2020. *MMWR Morb Mortal Wkly Rep*. 2020;69(37):1296-1299. doi:10.15585/mmwr.mm6937a4

11. Telford CT, Bystrom C, Fox T, et al. COVID -19 Infection Prevention and Control Adherence in Long-Term Care Facilities, Atlanta, Georgia. *J Am Geriatr Soc*. 2021;69(3):581-586. doi:10.1111/jgs.17001

12. Holmdahl I, Kahn R, Hay JA, Buckee CO, Mina MJ. Estimation of Transmission of COVID-19 in Simulated Nursing Homes With Frequent Testing and Immunity-Based Staffing. *JAMA Netw Open*. 2021;4(5):e2110071. doi:10.1001/jamanetworkopen.2021.10071

13. Nguyen LKN, Howick S, McLafferty D, et al. Evaluating intervention strategies in controlling coronavirus disease 2019 (COVID-19) spread in care homes: An agent-based model. *Infect Control Hosp Epidemiol*. 2021;42(9):1060-1070. doi:10.1017/ice.2020.1369

14. Smith DR, Duval A, Pouwels KB, et al. *Optimizing COVID-19 Surveillance in Long-Term Care Facilities: A Modelling Study*. Epidemiology; 2020. doi:10.1101/2020.04.19.20071639

15. Vilches TN, Nourbakhsh S, Zhang K, et al. Multifaceted strategies for the control of COVID-19 outbreaks in long-term care facilities in Ontario, Canada. *Prev Med*. 2021;148:106564. doi:10.1016/j.ypmed.2021.106564

16. Adult Social Care Rapid Testing Fund. GOV.UK. Accessed October 7, 2022. https://www.gov.uk/government/publications/adult-social-care-rapid-testing-fund

17. Adult Social Care Infection Control Fund. GOV.UK. Accessed July 26, 2022. https://www.gov.uk/government/publications/adult-social-care-infection-control-fund

18. Gray KL, Birtles H, Reichelt K, James IA. The experiences of care home staff during the COVID-19 pandemic: A systematic review. *Aging Ment Health*. 2022;26(10):2080-2089. doi:10.1080/13607863.2021.2013433

19. Pétré B, Paridans M, Gillain N, et al. Factors influencing the adoption and participation rate of nursing homes staff in a saliva testing screening programme for COVID-19. *PloS One*. Published online June 30, 2022. doi:10.1371/journal.pone.0270551

20. Pétré B, Paridans M, Gillain N, et al. Acceptability of Community Saliva Testing in Controlling the COVID-19 Pandemic: Lessons Learned from Two Case Studies in Nursing Homes and Schools. *Patient Prefer Adherence*. 2022;16:625-631. doi:10.2147/PPA.S349742

21. Walker, J. Implementing COVID-19 infection prevention and control measures in long-term care settings. *Nurs Older People*. Published online April 5, 2022. doi:10.7748/nop.2022.e1384

22. Nyashanu M, Pfende F, Ms E. Triggers of mental health problems among frontline healthcare workers during the COVID-19 pandemic in private care homes and domiciliary care agencies: Lived experiences of care workers in the Midlands region, UK. *Health Soc Care Community*. Published online February 2022. doi:10.1111/hsc.13204

23. Tulloch J, Micocci M, Buckle P, et al. Enhanced lateral flow testing strategies in care homes are associated with poor adherence and were insufficient to prevent COVID-19 outbreaks: results from a mixed methods implementation study. *Age Ageing*. Published online November 10, 2021. doi:10.1093/ageing/afab162

24. Kierkegaard P, Micocci M, McLister A, et al. Implementing lateral flow devices in long-term care facilities: experiences from the Liverpool COVID-19 community testing pilot in care homes- a qualitative study. *BMC Health Serv Res*. Published online October 25, 2021. doi:10.1186/s12913-021-07191-9

25. Oldfield B, DeCosta S, Petterson L, Lagarde S, Olson D. A Blueprint for Community Health Center and Nursing Home Partnership: Testing for COVID-19 among Residents and Staff at Long-term Care Facilities. *J Health Care Poor Underserved*. Published online 2021. doi:10.1353/hpu.2021.0001

26. Micocci M, Gordon A, Allen A, et al. COVID-19 testing in English care homes and implications for staff and residents. *Age Ageing*. Published online May 5, 2021. doi:10.1093/ageing/afab015

27. White E, Wetle T, Reddy A, Baier R. Front-line Nursing Home Staff Experiences During the COVID-19 Pandemic. *J Am Med Dir Assoc*. Published online January 2021. doi:10.1016/j.jamda.2020.11.022

28. Ouslander J, Grabowski D. COVID-19 in Nursing Homes: Calming the Perfect Storm. *J Am Geriatr Soc*. Published online October 2020. doi:10.1111/jgs.16784

29. Wasserman M, Ouslander J, Lam A, et al. Editorial: Diagnostic Testing for SARS-Coronavirus-2 in the Nursing Facility: Recommendations of a Delphi Panel of Long-Term Care Clinicians. *J Nutr Health Aging*. Published online 2020. doi:10.1007/s12603-020-1401-9

30. Gordon A, Goodman C, Achterberg W, et al. Commentary: COVID in care homes-challenges and dilemmas in healthcare delivery. *Age Ageing*. Published online August 24, 2020. doi:10.1093/ageing/afaa113

31. COVID-19 and policies for care homes in the first wave of the pandemic in European welfare states: Too little, too late? - Mary Daly, Margarita León, Birgit Pfau-Effinger, Costanzo Ranci, Tine Rostgaard, 2022. Accessed July 26, 2022. https://journals.sagepub.com/doi/full/10.1177/09589287211055672

32. Social care 360: workforce and carers. The King's Fund. Accessed October 7, 2022. https://www.kingsfund.org.uk/publications/social-care-360/workforce-and-carers

33. Recruitment and retention. Accessed October 7, 2022. https://www.skillsforcare.org.uk/adult-social-care-workforce-data/Workforce-intelligence/publications/Topics/Recruitment-and-retention.aspx

34. UKHSA. Personal communication.

35. Fisman DN, Bogoch I, Lapointe-Shaw L, McCready J, Tuite AR. Risk Factors Associated With Mortality Among Residents With Coronavirus Disease 2019 (COVID-19) in Long-term Care Facilities in Ontario, Canada. *JAMA Netw Open*. 2020;3(7):e2015957. doi:10.1001/jamanetworkopen.2020.15957

36. Krutikov M, Palmer T, Tut G, et al. Prevalence and duration of detectable SARS-CoV-2 nucleocapsid antibodies in staff and residents of long-term care facilities over the first year of the pandemic (VIVALDI study): prospective cohort study in England. *Lancet Healthy Longev*. 2022;3(1):e13-e21. doi:10.1016/S2666-7568(21)00282-8

37. Krutikov M, Palmer T, Donaldson A, et al. Study Protocol: Understanding SARS-Cov-2 infection, immunity and its duration in care home residents and staff in England (VIVALDI). *Wellcome Open Res*. 2021;5:232. doi:10.12688/wellcomeopenres.16193.2

38. Krutikov M, Hayward A, Shallcross L. Spread of a Variant SARS-CoV-2 in Long-Term Care Facilities in England. *N Engl J Med*. 2021;384(17):1671-1673. doi:10.1056/NEJMc2035906

39. Krutikov M, Palmer T, Tut G, et al. Incidence of SARS-CoV-2 infection according to baseline antibody status in staff and residents of 100 long-term care facilities (VIVALDI): a prospective cohort study. *Lancet Healthy Longev*. 2021;2(6):e362-e370. doi:10.1016/S2666-7568(21)00093-3

40. Shrotri M, Krutikov M, Palmer T, et al. Vaccine effectiveness of the first dose of ChAdOx1 nCoV-19 and BNT162b2 against SARS-CoV-2 infection in residents of long-term care facilities in England (VIVALDI): a prospective cohort study. *Lancet Infect Dis*. 2021;21(11):1529-1538. doi:10.1016/S1473-3099(21)00289-9

41. Competition & Markets Authority. *Guidance: Care Homes Market Study: Summary of Final Report*.; 2017.

42. The World Cafe Community Foundation. World Cafe Method. The World Cafe. Published July 4, 2015. Accessed July 27, 2022. http://theworldcafe.com/key-concepts-resources/world-cafe-method/

43. UKHSA. *COVID-19 Testing in Adult Social Care*.; 2022.

44. COVID-19 Datastore Reference Library. Accessed October 7, 2022. https://data.england.nhs.uk/covid-19/

45. UCL. Data Safe Haven (DSH). Information Services Division. Published February 14, 2018. Accessed October 7, 2022. https://www.ucl.ac.uk/isd/services/file-storage-sharing/data-safe-haven-dsh

46. Gomes M, Grieve R, Nixon R, Ng ESW, Carpenter J, Thompson SG. METHODS FOR COVARIATE ADJUSTMENT IN COST-EFFECTIVENESS ANALYSIS THAT USE CLUSTER RANDOMISED TRIALS: METHODS FOR COVARIATE ADJUSTMENT IN CEA OF CRTs. *Health Econ*. 2012;21(9):1101-1118. doi:10.1002/hec.2812

47. Achana F, Gallacher D, Oppong R, et al. Multivariate Generalized Linear Mixed-Effects Models for the Analysis of Clinical Trial–Based Cost-Effectiveness Data. *Med Decis Making*. 2021;41(6):667-684. doi:10.1177/0272989X211003880

48. NHS Improvement. *National Cost Collection: National Schedule of NHS Costs - Year 2019-20 - NHS Trust and NHS Foundation Trusts.*; 2020.

49. Jones, Karen C., Burns, Amanda. *Unit Costs of Health and Social Care 2021*. Personal Social Services Research Unit; 2021. doi:10.22024/UNIKENT/01.02.92342

50. Public involvement. Health Research Authority. Accessed October 7, 2022. https://www.hra.nhs.uk/planning-and-improving-research/best-practice/public-involvement/

51. Skivington K, Matthews L, Simpson SA, et al. A new framework for developing and evaluating complex interventions: update of Medical Research Council guidance. *BMJ*. 2021;374. doi:10.1136/bmj.n2061

52. Moore G, Audrey S, Barker M, et al. Process evaluation of complex interventions: Medical Research Council guidance. *BMJ*. Published online March 19, 2015. doi:10.1136/bmj.h1258

53. May C. Towards a general theory of implementation. *Implement Sci*. 2013;8(1):18. doi:10.1186/1748-5908-8-18

54. Murray E, Treweek S, Pope C, et al. Normalisation process theory: a framework for developing, evaluating and implementing complex interventions. *BMC Med*. 2010;8(1):63. doi:10.1186/1741-7015-8-63

55. Rutter H, Savona N, Glonti K, et al. The need for a complex systems model of evidence for public health. *The Lancet*. 2017;390(10112):2602-2604. doi:10.1016/S0140-6736(17)31267-9

56. Clarke, V, Braun, V, Hayfield, N. *Thematic Analysis. Qualitative Psychology: A Practical Guide to Research Methods.* SAGE Publications; 2015.

57. Shaw R, Larkin M, Flowers P. Expanding the evidence within evidence-based healthcare: thinking about the context, acceptability and feasibility of interventions. *Evid Based Med*. Published online December 2014. doi:10.1136/eb-2014-101791

58. Williams L, MacDonald B, Rollins L, et al. Sharing positive behavior change made during COVID-19 lockdown: A mixed-methods coproduction study. *Health Psychol Off J Div Health Psychol Am Psychol Assoc*. Published online October 2021. doi:10.1037/hea0001130

59. Flowers P, Riddell J, Park C, et al. Preparedness for use of the rapid result HIV self-test by gay men and other men who have sex with men (MSM): a mixed methods exploratory study among MSM and those involved in HIV prevention and care. *HIV Med*. Published online April 2017. doi:10.1111/hiv.12420

60. Cane J, O'Connor D, Michie S. Validation of the theoretical domains framework for use in behaviour change and implementation research. *Implement Sci*. 2012;7(1):37. doi:10.1186/1748-5908-7-37

61. May CR, Albers B, Bracher M, et al. Translational framework for implementation evaluation and research: a normalisation process theory coding manual for qualitative research and instrument development. *Implement Sci*. 2022;17(1):19. doi:10.1186/s13012-022-01191-x

Appendix A: VIVALDI-CT outbreak escalation decision algorithm

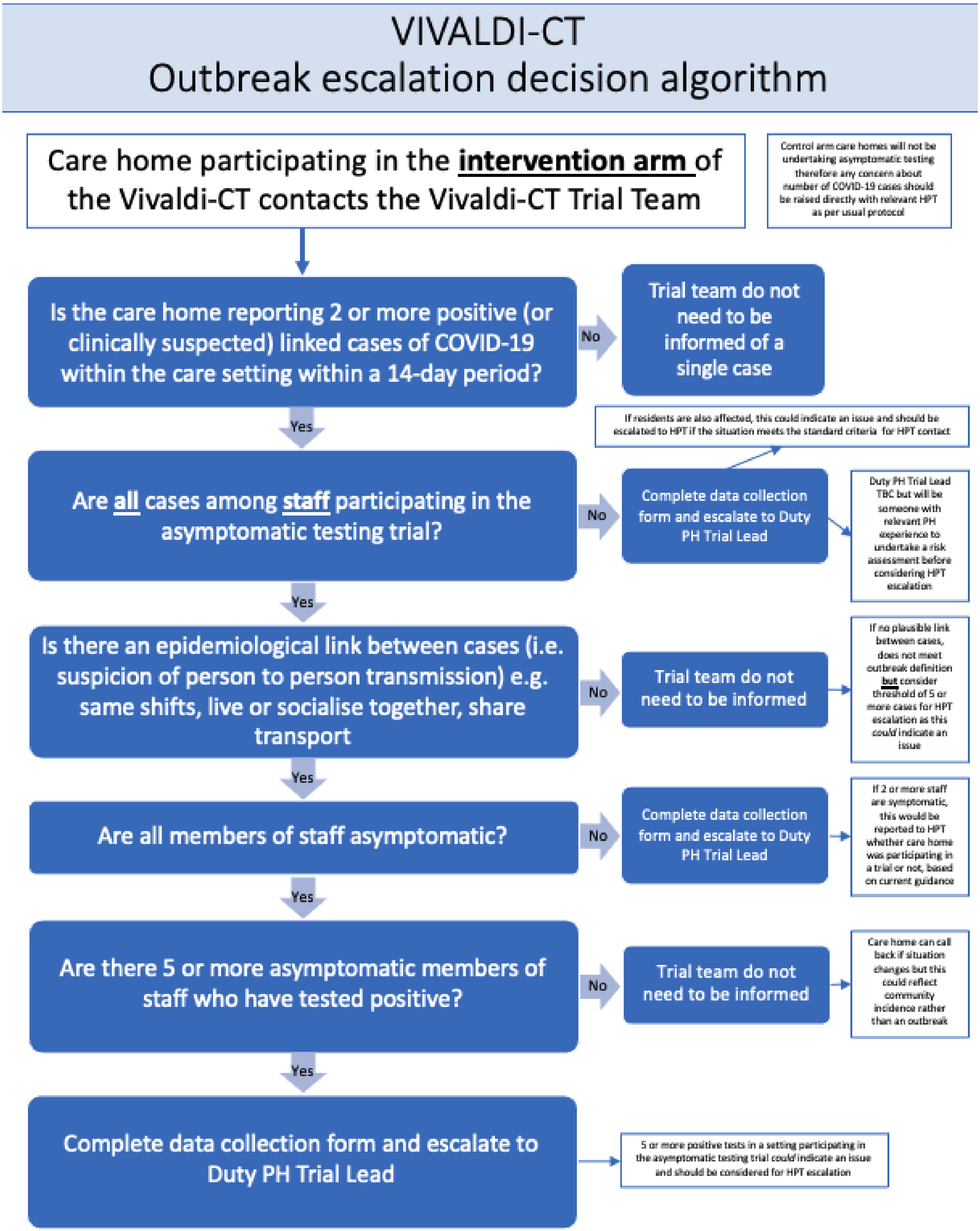

Supplement: S1 File — (PDF) [file pone.0324908.s006.pdf]
