# Supplementary material for: VIVALDI-CT shaping care home COVID-19 testing policy: A pragmatic cluster randomised controlled trial of asymptomatic testing compared to standard care in care home staff
Source: PLoS One. 2025 Jul 2;20(7):e0324908. doi: 10.1371/journal.pone.0324908 (PMC12221029; doi:10.1371/journal.pone.0324908)
Supplement: S2 File — (PDF) [file pone.0324908.s007.pdf]

RANDOMISATION PROTOCOL FOR VIVALDI-CT

Shaping care home COVID-19 testing policy: A pragmatic cluster randomised controlled trial of an intervention to promote regular, asymptomatic testing in care home staff

Written by Oliver Stirrup on 8th December 2022

Version: V0.4.

1 INTRODUCTION

This protocol sets out how the care homes participating the VIVALDI clinical trial will be assigned to intervention (asymptomatic testing of staff) or control (testing following national guidelines) conditions. This protocol was written with reference to the VIVALDI-CT protocol version 2.0 (28/11/2022).

2 RESPONSIBILITIES

This randomisation protocol is for use by the statisticians assigning randomisation status for care homes participating in the study.

Randomisation will be 1:1 to intervention (asymptomatic testing of staff) and control (testing following national guidelines) conditions. The randomisation process will be conducted separately for each care home Provider joining the study. This will ensure stratification of randomisation by Provider, and will also allow a staggered start for different Providers as they join the study. Randomisation may also be conducted separately for batches of homes joining the study at the same time from a single Provider, if it is not possible to confirm all participating homes from that Provider at a single point in time. Restricted randomisation will be used to ensure balance of assignment within regions for each Provider, and such that the mean number of residents per home does not greatly differ between intervention and control sites. More specifically, covariate-by-covariate constrained randomization will be used.

For each Care Home Provider, the Senior Statistician will create a 1:1 table of the unique CQC-ID codes with a new anonymised ID code. The Trial Statistician will create randomisation assignments based on this that will be output to a CSV file, and the Senior Statistician will then reveal the randomisation output by CQC-ID. Intervention and control conditions will not be blinded once linked back to CQC-ID, and assigned status will be communicated to individual care homes via each Provider.

3 TESTING

The program will undergo testing to ensure it is functioning as expected. This will include:

1. Checking there are similar numbers randomised to each group (within +/- 1 for each Provider)
2. Checking that the mean number of residents per home is balanced between control and intervention arms within the tolerance specified for each Provider.
3. Checking that the number of homes per region is balanced between control and intervention arms within the tolerance specified for each Provider.
4. Checking that the proportion of acceptable randomisations is not overly restricted for each Provider (i.e. >20% of all possible randomisations acceptable according to restriction conditions)
5. Checking that there are no pairings of homes for which the two homes are either never or always allocated to the same trial arm within the acceptable randomisations (unless there are only two homes within a given region for a Provider, in which case the two homes would always be allocated to different trial arms). We will also review the allocation if any pair of homes is allocated to either the same trial arm for <10% or >90% of acceptable randomisations.

Testing will be conducted using dummy datasets prior to the start of the trial, and for each individual randomisation list produced for each Provider taking part in the trial.

4 IMPLEMENTING THE RANDOMISATION LIST

The randomisation list for each Provider will be produced and checked by the Trial Statistician, and the Senior Statistician will also review output to check points 1-3 listed above.

The final randomisation list for each Provider will be stored within the eTMF. The Trial Manager will pass the randomisation list to each Provider once created, and the Provider will communicate the intervention assignments to individual participating care homes.

Randomisation assignment will not be blinded once linked to the CQC-ID codes, as this would not be possible given that data processing by trial arm will be needed throughout the trial to evaluate data collection and testing implementation.

5 TECHNICAL DETAILS OF COVARIATE-BY-COVARIATE CONSTRAINED RANDOMIZATION

Covariate-by-covariate constrained randomisation will be implanted using the ‘cvrcov’ function in the cvcrand package for R (v0.1.0, Yu et al., 2019, 2020). This will be implemented separately for each Provider. If a Provider has an odd number of care homes (N) participating in the study, then whether (N-1)/1 or (N+1)/1 homes are assigned to the intervention condition will be determined using pseudorandom number generation (with 0.5 probability for either option) prior to use of the cvcrand function.

The absolute mean difference in the mean number of residents within each arm will be restricted to be within 1 SD of the home size observations (i.e. by number of residents) within that Provider.

Regions of England will be group as ‘North’ (North East, North West, and Yorkshire and Humber), ‘Midlands’ (East Midlands, West Midlands, East of England), ‘South’ (South East and South West) and ‘London’. The maximum within-Provider within-region split between trial arms will be 60:40, unless a larger proportional split is necessary based on a small number of sites (i.e. three sites in a given region will be distributed 2:1 across trial arms). This will be enforced by setting the absolute mean difference of the proportion of sites within a given region (n) between the two trial arms to the maximum of:
● 0.6*n_region/((N_homes-1)/2) - 0.4*n_region/((N_homes+1)/2)
And
● 2*(n_region+N_homes) / (N_homes^2-1)
This translates the maximum allowable difference specified into the format required for the statistical software used.

The Trial Statistician has pre-generated a list of random number seeds, not shared with the rest of the trial team, that will be used for each new Provider undergoing randomisation for the study.

6 ADJUSTMENT OF RANDOMISATION PLAN FOLLOWING TRIAL INITIATION

If a batch of three homes or fewer joins the trial, then simple randomisation will be used. For one home, a single Bernoulli variable will be generated. For two or three homes, sites for the intervention will be sampled without replacement. For three homes, whether one or two homes are allocated the intervention will be determined randomly.

REFERENCES

Yu H, Li F, Gallis J, Turner E (2020). cvcrand: Efficient Design and Analysis of Cluster Randomized Trials. R package version 0.1.0, <https://CRAN.R-project.org/package=cvcrand>.

Yu H, Li F, Gallis JA and Turner EL. cvcrand: A Package for Covariate-constrained Randomization and the Clustered Permutation Test for Cluster Randomized Trials.
The R Journal 2019 11:2, pages 191-204.
