# Supplementary material for: VIVALDI-CT shaping care home COVID-19 testing policy: A pragmatic cluster randomised controlled trial of asymptomatic testing compared to standard care in care home staff
Source: PLoS One. 2025 Jul 2;20(7):e0324908. doi: 10.1371/journal.pone.0324908 (PMC12221029; doi:10.1371/journal.pone.0324908)
Supplement: S3 File — (PDF) [file pone.0324908.s008.pdf]

VIVALDI-CT

Shaping care home COVID-19 testing policy: A pragmatic cluster randomised controlled trial of asymptomatic testing compared to standard care in care home staff.

Protocol version: V3.0 dated 04.01.2023
IRAS ID: 320847

STATISTICAL ANALYSIS PLAN (SAP)

VERSION 1.1, 7TH JUNE 2023

**Authorised by:**	**Signature**	**Date**
Prof Laura Shallcross, Chief Investigator<br>Email: l.shallcross@ucl.ac.uk	______________	______________
Prof Andrew Copas, Oversight Statistician<br>Email: a.copas@ucl.ac.uk	______________	______________

First draft prepared by:
Oliver Stirrup, Trial Statistician
Email: oliver.stirrup@ucl.ac.uk

VIVALDI-CT SAP version 1.1, 7th June 2023

Table of contents

1 ABBREVIATIONS 4
2 DEFINITIONS 4
3 BACKGROUND AND DESIGN 5
4 OUTCOME MEASURES 6
4.1 Primary outcome 6
4.2 Secondary outcomes 6
5 DATA........ 7
5.1 Data collection and management 7
5.2 Data completion and schedule 8
5.3 Analysis dataset 9
5.4 Data verification 9
5.5 Data coding........ 9
5.6 Coding of primary and secondary outcomes 10
5.6.1 Primary outcome 10
5.6.2 Secondary outcomes 10
5.7 Coding of adjustment variables 12
5.7.1 Care home provider........ 12
5.7.2 Care home region 12
5.7.3 Care home size 12
5.8 Coding of calendar time 13
6 SAMPLE SIZE ESTIMATION 13
7 ANALYSIS PRINCIPLES 13
7.1 Intention-to-treat (ITT) or per-protocol? 13
7.2 Confidence Intervals and P-Values 13
7.3 Baseline comparability 14
7.4 Adjustment for design, baseline and contextual factors 14
7.5 Missing data 14
7.6 Summarising models 14
7.7 Descriptive summaries 15
8 ANALYSIS DETAILS 15
8.1 Recruitment and intervention implementation 15
8.2 Baseline Characteristics 15
8.3 Analysis Methods 15
8.3.1 Random effects structures 15
8.3.2 Incidence outcomes 15
8.3.3 Outcomes expressed as a proportion 16
8.3.4 Calculation of marginal estimates and differences 16
8.3.5 Outbreak duration 16
8.4 Adjustment for baseline and contextual factors in analysis 16
8.5 Subgroup analyses 17
8.6 Regression diagnostics 17
9 INTERIM ANALYSES 17

9.1 Regular reporting to TSC-DMC 17
9.2 Formal interim analysis 17
10 UPDATE LOG 19
11 TABLES AND GRAPHS: 20
12 REFERENCES 25

1 ABBREVIATIONS

Acronyms	Meaning
A&E	Accident and Emergency
CAG	Confidentiality Advisory Group
CCTU	Comprehensive Clinical Trials Unit
COVID-19	Coronavirus disease 2019
CRF	Case Record Form
CQC	Care Quality Commission
DSH	Data Safe Haven
ED	Emergency department
HRA	Health Research Authority
ICD10	International Classification of Diseases 10th Revision
IRR	Incidence Rate Ratio
LFD	Lateral Flow Device
LRT	Likelihood ratio test
OR	Odds Ratio
PCR	Polymerase Chain Reaction
SARS-CoV-2	Severe acute respiratory syndrome coronavirus 2
SOP	Standard Operating Protocols
TMF	Trial Master File
TMG	Trial Management Group
TSC-DMC	Trial steering committee – data monitoring committee
UCL	University College London
UK HSA	UK Health Security Agency

2 DEFINITIONS

- **COVID-19** - the disease caused by the SARS-CoV-2 virus.
- **Care home** - in the UK, care homes provide accommodation, together with nursing or personal care, for persons who are or have been ill, who have or have had a mental disorder, who are disabled or infirm, or are or have been dependent on alcohol or drugs. They include homes with and without 24 h onsite nursing staff, known as residential and nursing homes, respectively. Care homes are expected to have largely exclusive staff. In the event that two nearby care home buildings, run by the same provider, share staff and local management then they will be considered as one home for the purposes of this trial, but will be considered as two if the staff and local management are largely distinct.
- **Provider -** UK care home groups

3 BACKGROUND AND DESIGN

Aim and objectives

The aim of the study is to determine whether regular asymptomatic testing in staff is a feasible, effective, and cost-effective strategy to reduce the impact of COVID-19 in care homes. Findings will shape policy across the UK for COVID-19 and inform wider strategies to prevent other respiratory viruses in care homes, such as influenza.

The primary outcome for the trial is the incidence of COVID-19 related hospital admissions in care home residents. There are a number of secondary outcomes selected to quantify the implementation of the intervention and the broader impact of the intervention on the functioning of care homes; these are specified later in the SAP.

This SAP specifies the study results to be included in the primary analysis publication (and preceding interim analysis for the trial), and does not cover the health economics, modelling or process evaluation analyses described in the overall study Protocol.

Population studied

This is a cluster randomised trial of care homes in England, with randomisation conducted at the level of each care home. Only care home staff are eligible to participate in the testing intervention. This includes temporary (agency) staff with no restrictions (e.g. including catering staff, administrative staff, maintenance staff), but not professionals visiting the care home such as GPs and health visitors. Pseudonymised data on SARS-CoV-2 testing will be extracted for staff from routine data sources for all participating care homes, and aggregate data on staff opting out of asymptomatic testing will be collected from care homes included in the intervention arm of the trial. Aggregate data on staff sickness absence will also be collected from care homes in both trial arms.

All residents at participating care homes are eligible for data collection and analysis of the outcomes specified, including COVID-19 associated hospital admissions, COVID-19 associated mortality and SARS-CoV-2 infections.

Study design

This study is a parallel group cluster randomised trial, with randomisation at the level of each care home.

Randomisation

Randomisation will be 1:1 to intervention (asymptomatic testing of staff) and control (testing following national guidelines) conditions. The randomisation process will be conducted separately for each care home Provider joining the study. Randomisation may also be conducted separately for batches of homes joining the study from a Provider, if it is not possible to confirm all participating homes from that Provider at a single point in time. Restricted randomisation will be used to ensure balance of assignment within regions for each Provider, and such that the mean number of residents per home does not greatly differ between intervention and control sites. This will be conducted

using the implemented using the ‘cvrcov’ function in the cvcrand package for R (v0.1.0)[1]. Full details are provided in a separate randomisation plan document.

Consent for participation in the study
The study has HRA CAG approval for the use of pseudonymised personal data without individual consent. However, staff and residents of participating care homes have the option to opt-out of the use of their individual-level data within the analysis dataset for this trial.

Blinding
Staff and residents of participating care homes will not be blinded to their intervention allocation, as staff of homes in the intervention arm will be asked to regularly self-test for SARS-CoV-2 using LFDs even when asymptomatic.

The Trial Statistician will remain blinded up until the final stages of analyses for the interim report. For the interim report, coding of data processing and analysis will first be completed for resident outcomes using a blinded intervention allocation code provided by the trial management team. Coding will then be completed for outcomes related to staff testing; this will also use the blinded intervention allocation code, but the results are likely to indicate which arm is the intervention. Finally, a summary will be created of staff opting out of the testing intervention, which will require access to unblinded data. The same analysis code will form the basis for reporting of results after completion of the study. The Oversight Statistician will remain blinded until completion of final analysis coding by the Trial Statistician.

The final decision regarding the coding of the primary outcome based on the different data sources for the final analysis will be taken based on a dataset with information on care homes and the associated intervention allocation for each resident removed (see section 5.6.1).

4 OUTCOME MEASURES

4.1 Primary outcome

- Incidence of COVID-19 related hospital admissions in residents, measured as events per 1000 person-years of follow-up over the duration of the trial.

4.2 Secondary outcomes

- Incidence rate of hospital admissions (all-cause) in residents for non-elective care, measured as events per 1000 person-years of follow-up over the duration of the trial.

- Incidence rate of COVID-associated mortality in residents, measured as events per 1000 person-years of follow-up over the duration of the trial*

- Incidence of all-cause mortality in residents, measured as events per 1000 person-years of follow-up over the duration of the trial.

- Testing uptake in staff, measured as proportion of staff at each home participating in testing during each week of the trial.

- Prevalence of SARS-CoV-2 among staff who test, measured as proportion of staff with positive test result among those with at least one test recorded during each week of the trial.

- Incidence rate of SARS-CoV-2 infections detected in residents, measured as events per 1000 person-years of follow-up over the duration of the trial.

- Incidence rate of home-level outbreaks as recorded by UKHSA, measured as events per 1000 days of follow-up over the duration of the trial.

- Duration of outbreaks, measured as days from first to last case within outbreaks occurring within the trial period.

- Incidence rate of care home closures due to outbreaks, measured as distinct closure events per 1000 days of follow-up over the duration of the trial.

- Proportion of staff per home who are off sick at each home during each week of the trial.

- Proportion of all shifts filled by agency staff at each home during each week of the trial.

- Composite incidence of COVID-19 related hospital admissions and COVID-19 related mortality* in residents, measured as events per 1000 person-years of follow-up over the duration of the trial. NOTE: this outcome is not listed in the current study protocol, but has been considered as a potential alternative Primary Outcome for the trial.

*COVID-associated mortality will be defined as death within 28 days of a positive SARS-CoV-2 test and/or COVID-19 recorded as primary or secondary cause of death on the death certificate (using ICD10 coding).

5 DATA

5.1 Data collection and management

Much of the data for analysis will be obtained from routinely collected healthcare information that is held within the UK COVID-19 Datastore (https://data.england.nhs.uk/covid-19/). This will include results of LFD and PCR tests for SARS-CoV-2, and information on hospital admissions and deaths. Data within the COVID-19 Datastore are linked to a pseudonymised ID code at the level of each individual, which can be linked to CQC IDs for participating care homes and associated staff or resident status.

Data on hospital admissions are linked to ICD10 diagnostic codes (which include specific codes for COVID-19) within the COVID-19 datastore. However, there is a lag of several months in the assignment of ICD10 codes to hospital admission data. As such, in order to allow timely monitoring of the primary

outcome and avoid the risk of omitting hospital admissions in residents, care providers will be asked to upload to the COVID Datastore weekly lists of COVID-associated hospital admissions occurring at participating care homes via a secure transfer process. These data will also be linked to pseudonymised ID codes for each individual, allowing comparison to routinely collected hospital admission data once available.

Data from the UK COVID-19 Datastore will be processed in the secure Foundry system (managed by a UK HSA Data Engineer), within which the relevant data records will be identified for participating care homes and individual level opt-out requests will be applied. Datasets for analysis will be exported from the Foundry to the UCL DSH secure environment. No data exports to the DSH will be made within the first 4 weeks of the trial intervention period in order to allow time for individual data opt outs to be processed. If someone submits an opt out request at any point during the trial, then their individual level data will be completely removed from all further analysis datasets exported to the UCL DSH. A new project-specific pseudo-ID code will be generated before the analysis data is exported to the UCL DSH, in order to prevent the possibility of direct linkage of individuals to further information in the source datasets hosted on Foundry.

In order to be able to express and analyse primary and secondary outcomes relating to residents as incidence rates, we require information on the denominator i.e. the total number of residents at each participating care home. This will be collected at weekly intervals in batch from each care provider. We will also collect the total number of staff and the number of staff who opt out of asymptomatic testing (in the intervention arm), staff sickness absence and employment of agency staff on a weekly basis for each home. Data on outbreak events (dates, size) will be obtained from the UKHSA Adult Social Care Team. These data will be transferred to the CCTU trial team by secure email transfer, and collated into datasets that will be uploaded to the UCL DSH for analysis.

Data processing and analysis will be conducted by the Trial Statistician within the UCL DSH following CCTU SOPs.

5.2 Data completion and schedule

A decision regarding the continuation of the intervention and collection of new data for the trial will be made by the TSC and trial funder, following guidance from the DMC, at the end of June 2023.

5.3 Analysis dataset

The dataset of information returned to CCTU directly by care home providers (e.g. including weekly information on resident and staff numbers for each home) will be 'locked' for analysis once all data transfer forms have been returned, data cleaning has been completed and all data queries are closed. A final frozen dataset will be created by the study Data Manager for inclusion within the statistical analysis.

Initial analysis datasets will be exported from the Foundry to the UCL DSH at the point of the interim analysis and 1 week after the cessation of the trial intervention. The latter will form the basis for an initial trial report for internal use and initial reporting to UK HSA. A final dataset will be exported from the Foundry to UCL DSH once ICD10 codes have been added to hospital admission and mortality data within the COVID-19 Datastore for the Trial period; this dataset will be used for the final trial report and publication.

5.4 Data verification

Basic data checks on the information provided directly by care home providers will be performed by the trial Data Manager and Trial Statistician periodically during the trial. Additional range, consistency and missing data checks will be performed when the datasets for analysis are constructed within the UCL DSH, as appropriate, before the statistical analysis is performed. All variables will be examined for unusual, outlying, unlabelled or inconsistent values.

Any problems with study data provided by care homes will be queried with the Trial Manager or Data Manager as appropriate. If possible, data queries will be resolved; although it is accepted that due to administrative reasons and data availability a small number of problems will continue to exist. These will be minimised.

Basic quality checks on routine health data collated through the Foundry platform will include:
-Confirmation that PCR and LFD test data are available for all participating care homes.
-Checking that individual-level staff testing data are consistent with the aggregate data collected from sites.
-Confirmation that resident mortality data span the time period of the trial.
-Confirmation that resident hospital admission data from each source span the time period of the trial.
-Comparison of site-provided data on resident hospital admissions and deaths against those collected from routine data.

Any inconsistencies identified will trigger checks of the coding for the data pipeline and of the source datasets within Foundry.

5.5 Data coding

For the information provided directly by care homes, specification of data coding is provided by the format of the spreadsheets used for data collection. Documentation of the processing and format of

routinely collected data within the Foundry is specified within a separate document stored in the trial master file.

5.6 Coding of primary and secondary outcomes

5.6.1 Primary outcome

Incidence of COVID-19 related hospital admissions in residents

Events are defined as admissions with a relevant ICD10 code (COVID hospitalisations to be defined as any hospital admission record with ICD10 code of 'U071' recorded, not limited to the primary code) and/or admissions in residents who test positive for COVID-19 within 24h following admission or in the 7 days before hospital admission. Both of these will be available as part of routinely collected data within the COVID-19 Datastore. However, there is a delay in the addition of ICD10 coding to hospital admission records and there is also a risk that hospital admissions of residents at participating care homes will be missed if their records cannot be linked to the relevant CQC ID. Participating care homes will be uploading trial-specific data on COVID-associated hospital admissions (based on the positive SARS-CoV-2 test criteria). This alone will form the basis for any interim analyses and for the initial trial analysis performed shortly after the intervention period has ended.

For the final trial analysis results using the trial-specific data uploaded by care-home providers will be checked against routinely collected data once the ICD10 coding for hospital admissions becomes available for the trial period. This comparison will be conducted using a dataset with information on care homes and associated intervention allocation for each resident removed. A decision will be made and documented regarding the final coding of the primary outcome (e.g. whether to use the home-supplied data alone or to also merge in the routinely collected data) by the trial team before the analysis models are re-run using the agreed definition. Routinely collected data will be used as well as the site supplied data if there are a non-negligible number of relevant events missing from the site-supplied data (this could be due to problems in obtaining site-supplied data on admissions, or a lack of testing for SARS-CoV-2 prior to arrival at hospital). If the discrepancy between data sources is small, then the site-supplied data will be used alone as some events may be linked incorrectly to a care home (e.g. if a resident has been discharged or moved to another care home following their most recent recorded SARS-CoV-2 test).

All analyses will use weekly numbers of care home residents provided directly by the providers to inform the 'exposure' variable of person-days at risk. If an individual has more than one admission meeting the definition of a COVID-associated hospital admission within 30 days, then only the first of these events will be counted for analyses.

5.6.2 Secondary outcomes

1: Incidence rate of hospital admissions (all-cause) in residents for non-elective care

Events are defined as all hospital admissions , as the proportion of elective admissions in this population is thought to be very low. As a sensitivity analysis, we will check the proportion of admissions with an A&E attendance within the previous 2 days. Both of these will be available as part of routinely collected data within the COVID-19 Datastore. Analyses will use weekly numbers of

care home residents provided directly by the providers to inform the ‘exposure’ variable of person-days at risk for each home.

If an individual has more than one hospital admission within 30 days, then only the first of these events will be counted for analyses.

2: Incidence rate of COVID-associated mortality in residents
Events are defined as death with a relevant ICD10 code (primary or secondary ICD10 code of ‘U071’) and/or within 28 days of a positive SARS-CoV-2 test. Both of these will be available as part of routinely collected data within the COVID-19 Datastore. However, there is a delay in the addition of ICD10 coding to mortality data. Therefore, mortality within 28 days of a positive test alone will form the basis for any interim analyses and for the initial trial analysis performed shortly after the intervention period has ended. In order to reduce the potential for reidentification of individual residents, only ‘month of death’ will be exported to the UCL DSH, with an accompanying indicator variable recording whether the death was within 28 days of a positive test. Analyses will use weekly numbers of care home residents provided directly by the providers to inform the ‘exposure’ variable of person-days at risk for each home.

3: Incidence of all-cause mortality in residents
Events are defined as any deaths of residents linked to a participating care home. This will be evaluated using routinely collected data within the COVID-19 Datastore. In order to reduce the potential for reidentification of individual residents, only ‘month of death’ will be exported to the UCL DSH. Analyses will use weekly numbers of care home residents provided directly by the providers to inform the ‘exposure’ variable of person-days at risk for each home.

4: Testing uptake in staff
This will be measured as proportion of staff at each home participating in testing during each week of the trial. The numerator for this calculation will be the total number of staff at each home with at least one SARS-CoV-2 test (LFD or PCR) recorded in each week of the study using routinely collected data, and the denominator will be the number of care home staff at the home as provided on a weekly basis by providers. For homes in the intervention arms, these data will also be compared to the recorded number of staff opting out of asymptomatic testing in each week of the trial.

5: Prevalence of SARS-CoV-2 among staff who test
This will be measured as the proportion of staff with positive test result among those with at least one test (LFD or PCR) recorded at each home during each week of the trial. This will be evaluated using routinely collected data from the COVID-19 Datastore.

6: Incidence rate of SARS-CoV-2 infections detected in residents
Events are defined as any positive SARS-CoV-2 tests (LFD or PCR) for residents linked to a participating care home. This will be evaluated using routinely collected data within the COVID-19 Datastore. Analyses will use weekly numbers of care home residents provided directly by the providers to inform the ‘exposure’ variable of person-days at risk for each home. After a positive test in any given individual, any further positive tests (e.g. confirmatory PCR following a LFD result) in

that individual will be ignored for the following 30 days, and this exclusion will be used to adjust down the person-days at risk for that care home as appropriate.

7: Incidence rate of home-level outbreaks
Events are defined as any outbreak recorded by the UKHSA Adult Social Care Team. Outbreaks are defined as 2 or more positive (or clinically suspected) cases of COVID-19 during a 14-day period.

8: Duration of outbreaks
Outbreak duration will be defined by the start and end dates as recorded by the UKHSA Adult Social Care Team.

9: Incidence rate of care home closures due to outbreaks
Data on the occurrence and dates of care home closures due to outbreaks will be collected from care home Providers.

10: Proportion of staff per home off sick
This will be measured as the proportion of staff with any sickness absence at each care home during each week of the trial. The numerator and denominator for calculation of this proportion are included in the weekly aggregate data collected directly from care home providers.

11: Proportion of all shifts filled by agency staff
This will be recorded for each care home during each week of the trial. The numerator and denominator for calculation of this proportion are included in the weekly aggregate data collected directly from care home providers.

12: *Composite incidence of COVID-19 related hospital admissions and mortality in residents*
This will be coded as per the Primary Outcome and Secondary Outcome 2. This will be analysed as a 'time-to-event' type outcome, with a single event considered to have occurred if COVID-linked hospitalisation is followed by COVID-associated death in any given person.

5.7 Coding of adjustment variables

5.7.1 Care home provider
This is a categorical variable that will be used for stratification of randomisation and adjustment of analysis models. A breakdown of outcomes by care home provider will not be made public.

5.7.2 Care home region
Regions of England will be group as 'North' (North East, North West, and Yorkshire and Humber), 'Midlands' (East Midlands, West Midlands, East of England), 'South' (South East and South West) and 'London', comprising a simplification of the standard 'regions of England' classification.

5.7.3 Care home size
Care home size will be defined as the number of residents for each care home at entry to the trial.

5.8 Coding of calendar time

Analyses will mostly be conducted with calendar time divided into 'study weeks', running from Monday-Sunday for all sites. Analyses of mortality will be conducted according to calendar month.

6 SAMPLE SIZE ESTIMATION

The original sample size calculation for the study was as follows:

> "Based on observational data from the VIVALDI study we found that over the 3-month period of January-March 2022 1.8% of residents had a COVID-19 related hospital admission, and the Intra-Cluster Correlation (ICC) across homes was 0.003 (95% CI 0.000-0.007). We assume that we will observe a cumulative incidence of around 3.0% in the trial, which would require a trial duration of 5-6 months if the incidence rate is similar to that in winter 2021/2022, in combination with a conservative ICC value of up to 0.01 (higher in line with the higher cumulative incidence compared to 3 months), and an average care home size of 35 residents with coefficient of variation in size of 0.5. With a total of 280 homes randomised 1:1 to trial arms and taking the usual two-sided test at 5% significance level, the design provides 84% power to detect a reduction in COVID-19 related admissions due to intervention to 1.9% (relative risk 0.63)."

However, in this trial design the number of homes can be 'traded off' against the cumulative incidence of primary outcome in the control arm, which could be increased by allowing a longer trial duration. The interim analysis report shared with the DMC will include power calculations relating to continuation of the trial based on the recruited number of care homes and possible incidence rates of the primary outcome.

7 ANALYSIS PRINCIPLES

7.1 Intention-to-treat (ITT) or per-protocol?

The main analyses for the primary and secondary outcomes will be carried out on an 'intention-to-treat' (ITT) basis according to the allocated trial arm (i.e. regardless of the home-level performance in implementing the intervention as planned). However, if a Provider or individual care home entirely drops out of the trial and provides no further data then we will consider the relevant homes to be no longer under follow-up for the study.

7.2 Confidence Intervals and P-Values

All confidence intervals will be 95% and two-sided. Statistical tests will use a two-sided *p* value cut-off of 0.05 for statistical significance, with likelihood ratio tests (LRTs) used to calculate *p* values for intervention effects (as these have better properties than Wald-type tests for non-normal models). Quadrature points for maximum likelihood estimation of generalised linear mixed effects models will be incremented until stability of parameter estimates and log-likelihood are achieved to ensure the validity of LRTs. The significance level of secondary outcomes will not be adjusted for multiple testing. Nor will the significance level be adjusted to account for the planned interim analysis, as there is not a formal stopping rule and cessation of the trial would only be expected in the event of very strong evidence.

7.3 Baseline comparability

Baseline characteristics of participating care homes will be summarised by trial intervention arm, as indicated in the draft Table 1.

7.4 Adjustment for design, baseline and contextual factors

We will account for the structure of the data with random effect terms for each care home.

Analysis models for primary and secondary outcomes will include adjustment for care home provider, region and size (in terms of the number of residents) at entry to the study, and will also include adjustment for calendar time.

7.5 Missing data

For analyses of routinely collected data obtained through the COVID-19 Datastore, results will be presented for data 'as recorded' within the exported dataset (e.g. including all-cause hospital admissions and mortality for residents, and testing data in residents and staff). For the primary outcome of COVID-19 associated hospital admission, results using the trial-specific data uploaded by care-home providers will be checked against routinely collected data once the ICD10 coding for hospital admissions becomes available for the trial period.

The trial team will make every effort to obtain the data on time-updated resident numbers necessary for modelling of the incidence outcomes among residents (hospitalisations, deaths, positive SARS-CoV-2 tests), which will be included in the analyses on a per week per care home basis. If data are missing for any isolated weeks at any given site, then we will consider interpolation to impute vales based on the available data for adjacent weeks. Interpolation will also be considered for the weekly total number of staff at each care home, required for the secondary outcome of testing uptake.

The trial team will also endeavour to obtain data required for the analysis of the proportion of staff with sickness absence per week and the proportion of shifts filled by agency staff. If any required variables are missing for a care home, then these outcomes will be considered to be missing for that home for that week; using an appropriate mixed effects model for analysis, this can be considered as implicit imputation of any missing outcome data based on the available information.

There will not be any missing data for adjustment variables, as these are required for the randomisation process for any care home to be included in the trial.

7.6 Summarising models

For the incidence rate outcomes (including the primary outcome), intervention effect estimates will be presented as incidence rate ratios (IRR) and 95% CIs (generated using the Wald method) comparing the intervention to the control arm. The difference between arms will also be estimated and presented as the incidence rate difference (IRD) with 95% CIs in the final analysis.

For the outcomes expressed as a proportion (e.g. proportion of staff testing per week), along with the intervention OR, the estimated marginal proportion obtained from the mixed effects logistic regression model will be reported for each trial arm with 95% CI, and the difference between the proportions in the trial arms will be calculated with 95% CIs in the final analysis.

We do not plan to apply any small sample corrections, given the target sample size of 280 care homes.

7.7 Descriptive summaries

Whilst formal statistical analysis of the primary and secondary outcomes will only be conducted as specified, additional descriptive summaries will also be provided (planned dummy tables are included in the SAP Section 10).

8 ANALYSIS DETAILS

8.1 Recruitment and intervention implementation

Details will be provided of the number of care homes recruited to the trial and the duration of their involvement.

Implementation of the trial intervention will be evaluated through the secondary outcome of testing uptake among staff, and a descriptive summary will also be presented of the number of staff opting out of asymptomatic testing at care homes within the intervention arm of the trial.

8.2 Baseline Characteristics

Baseline characteristics of participating care homes will be summarised by trial arm. Summary measures for the baseline characteristics of each phase will be presented as median and interquartile range for continuous variables and frequencies and percentages for categorical variables.

8.3 Analysis Methods

Analyses will be conducted using Stata V17 unless specified otherwise.

8.3.1 Random effects structures

All random effects models will include a random intercept term per care home. All random effects will be specified as normally distributed.

8.3.2 Incidence outcomes

Incidence outcomes relating to hospitalisation and positive SARS-CoV-2 tests at the level of individual care home residents (including the primary outcome) will be analysed on a weekly basis and expressed per 1000 person-years of follow-up. Incidence outcomes of COVID-19 associated and all-cause mortality will be analysed on a monthly basis (as only month of death will be exported for analysis) and expressed per 1000 person-years of follow-up. Mixed effects negative binomial models

will be used for these outcomes, using weekly or monthly event counts as the outcome variable, with cluster-robust standard errors. The overall incidence rate calculated over the total person-days of follow-up within each trial arm will also be reported. A descriptive evaluation of between-site variation will be provided including the median, IQR and range of observed overall incidence rates for each care home.

Incidence outcomes at the level of each care home (e.g. outbreak events and closures) will be analysed on a weekly basis and expressed per 1000 days of follow-up. Poisson models will be used for these outcomes (selected rather than negative binomial models given the likely much lower event count for this outcome). The overall incidence rate calculated over the total follow-up of care homes within each trial arm will also be reported. A descriptive evaluation of between-site variation will be provided including a summary of the number of outbreak events and closures for each individual care home.

Data for incidence outcomes in the first 2 weeks of involvement for each site will be considered to represent a transition period for the intervention sites, and so will be omitted from analysis.

Exploratory graphical summaries of incidence outcomes over time for each site will also be produced. Marginal difference in incidence rate between the intervention and control arms will be calculated and reported.

8.3.3 Outcomes expressed as a proportion

For the outcomes expressed as a proportion (e.g. proportion of staff testing per week), analysis will be conducted using binomial form mixed effects logistic regression applied to weekly data from each home, with cluster-robust standard errors. Marginal proportions from the fitted models will be reported for the intervention and control arms, with the marginal risk difference also calculated and reported. A descriptive evaluation of between-site variation will be provided including the median, IQR and range of observed mean weekly values across sites.

8.3.4 Calculation of marginal estimates and differences

Marginal proportions and risk differences, and differences in incidence rates will be calculated using the ‘margins’ command in *Stata*. This command estimates hypothetical marginal value were all sites either under the control condition or under the intervention, based on the covariable values present within the dataset as a whole and integrating over variability associated with random effect terms. We will use the ‘unconditional’ standard error option for the margins command in order to account for sampling of covariable values from an underlying population of interest.

8.3.5 Outbreak duration

Outbreak duration will be analysed using a mixed effects negative binomial model.

8.4 Adjustment for baseline and contextual factors in analysis

For all incidence and proportion outcomes, models will be adjusted by care home provider, region, care home size and calendar time. Care home provider and region will be included as categorical variables. ‘Independent’ care homes, and homes that are the only home from a provider participating in the trial, will form a separate category for the care home provider adjustment factor.

Care home size and calendar time will be included as continuous variables for most outcomes, allowing for potentially non-linear adjustment using a 5 knot restricted cubic spline[2]. The fitted relationship for these analyses will be reported in the primary study report. For mortality outcomes, adjustment for calendar time will use a categorical variable for calendar month, given that death events are only available with 'month of death' recorded.

For adjustments related to both calendar time and care home size, a simpler relationship (e.g. linear adjustment) will be considered if the spline model does not converge or shows very large standard errors for estimated coefficients.

8.5 Subgroup analyses

We will define an implementation score based on the frequency and proportion of staff testing at each home based on data the homes provide, which may vary over time. In exploratory analysis we will assess whether the primary outcome is associated with this implementation score within the intervention arm and express the effect of the intervention relative to control arm for different levels of implementation. This analysis will be based on the same regression method as used for the primary analysis.

Using interaction terms, we will explore whether the effect of the intervention on the primary outcome differed between time periods defined by the national recommendations for testing in the routine care arm, should these change. To date, we would evaluate the periods before 3 April 2023 (symptomatic testing in residents and staff plus outbreak testing) and 3 April and beyond (symptomatic testing only in residents who are eligible for covid therapeutics + outbreak testing). To allow better consideration of the likely intervention impact across the whole care home sector we will also explore whether the intervention effect differs according to care home size, and other characteristics such as proportion of temporary staff.

8.6 Regression diagnostics

We shall check for model stability primarily through examination of standard errors for covariates, with large standard errors or convergence problems indicating a problem of model instability.

9 INTERIM ANALYSES

9.1 Regular reporting to TSC-DMC

Regular reporting to the TSC-DMC will be limited to information on the recruitment and randomisation of care homes into the trial.

9.2 Formal interim analysis

A single interim report will be produced after approximately 3 months of operating the intervention to allow an independent committee to assess the emerging benefit / risk ratio, and process outcomes such as the actual level of testing in both trial arms. This is scheduled for June 2023. This

report will include a table of baseline characteristics for recruited homes and a reduced subset of the primary and secondary outcomes of the trial (specified in the reduced Table 2 in Section 10).

We anticipate that there would only be a recommendation to stop the trial in the event of very strong evidence of benefit or harm, or if the recruitment and observed incidence rate of the primary outcome indicate that the trial would be inadequately powered to provide an answer regarding its main research objective within any realistic follow-up period. Due to the complexity of the planned analyses and time pressures, full conditional power analyses will not be included in the interim analysis report based on the data collected so far. However, the report will include updated power calculations based on the number of recruited homes and possible trial extension durations with varying incidence rates for the primary outcome in the control arm (informed by the incidence rate observed to date). Individual-level cumulative incidence estimates of the primary outcome will be estimated for comparison with the assumptions used for the initial sample size calculations.

The anticipated report contents are as follows:

Open report (shared with trial team and TSC)

1. Summary of recruitment and retention of homes
2. Baselinecharacteristics of homes by arm
3. Summary of completeness of data
4. Level of staff testing by arm
5. Primary outcome incidence and selected secondary outcomes (as in reduced version of Table 2) in control arm only
6. Power calculations for selected trial durations, given primary outcome incidence continues as in the trial thus far, or increases by a factor of 2, 5, or 10, reflecting a ‘wave’ or greater incidence in an Autumn/Winter period.
7. Individual-level cumulative incidence estimate of the primary outcome in the control arm.

Closed report (only shared with DMC)

1. Primary and key secondary outcomes by arm (as in reduced version of Table 2), with IRRs for incidence outcomes and aORs for binary outcomes for intervention vs control groups.

10 UPDATE LOG

Version	Change
1.1	NOTE: All version 1.1 changes made following review and feedback from DMC Chair.
1.1	Clarification added to '7.5 Missing data': "(e.g. including all-cause hospital admissions and mortality for residents, and testing data in residents and staff)"
1.1	Clarification added regarding use of Poisson models for some incidence outcomes (8.3.2): "(selected rather than negative binomial models given the likely much lower event count for this outcome)"
1.1	Clarification added regarding '8.3.3 Outcomes expressed as a proportion': "with the marginal risk difference also calculated and reported"
1.1	Cluster robust standard errors for models now specified in 8.3.2 and 8.3.3
1.1	New subsection added for clarity: '8.3.4 Calculation of marginal estimates and differences'.
1.1	Clarification added regarding adjustment for calendar month for outcomes at monthly level '8.4 Adjustment for baseline and contextual factors in analysis': "given that death events are only available with 'month of death' recorded"
1.1	Clarifications to '9.2 Formal interim analysis': "Baseline characteristics of homes by arm" and "Primary and key secondary outcomes by arm (as in reduced version of Table 2), with IRRs for incidence outcomes and aORs for binary outcomes for intervention vs control groups."
1.1	Addition to '9.2 Formal interim analysis': "Individual-level cumulative incidence estimate of the primary outcome in the control arm."
1.1	Addition of 'event counts' to version of Table 2 for interim report.

11 TABLES AND GRAPHS:

Table 1: Baseline Characteristics of the care homes by trial arm

	**Trial arm**	
	Control	Intervention
n care homes		
Region		
North		
Midlands		
South		
London		
Care home size (*n* residents)		
Care home type		
For profit		
Not for profit		
Number of permanent staff		
Type of care, by bed		
Nursing (%)		
Residential (%)		
Dementia (%)		
Bed occupancy (%)		
Ethnicity of staff		
Asian or Asian British (%)		
Black, Black British, Caribbean or African (%)		
Mixed or multiple ethnic groups (%)		
White British (%)		
White other (%)		
Other ethnic group (%)		
n outbreaks in 3 months prior to study entry		
Home in outbreak at study entry		
Disease control measures in place at study start		
Staff wearing masks at work		
Social distancing protocols for visitors		
Social distancing protocols for staff		
Cohorting of staff and residents		
Enhanced cleaning procedures		
Proportion of staff with ≥2 vaccine doses		
CQC rating		
Outstanding		
Good		
Requires improvement		
Inadequate		

Data presented as *n/N* (%) [aggregated over all care homes] or median (IQR).

Table 2: Summary and analyses of primary and secondary outcomes

	**Trial arm**		
	Control	Intervention	**Intervention vs control**
	**Median (IQR, range)**	**Median (IQR, range)**	
Weeks of participation per care home			—
Primary outcome	**Overall; Median (IQR, range) per home**	**Overall; Median (IQR, range) per home**	**IRR* (95% CI, *P*); marginal rate difference (95% CI)**
Incidence rate of COVID-19 associated hospital admissions per 1000 person-years†			
Secondary outcomes	**Overall; Median (IQR, range) per home**	**Overall; Median (IQR, range) per home**	**IRR* (95% CI, *P*) ; marginal rate difference (95% CI)**
Incidence rate of hospital admissions (all-cause) per 1000 person-years†			
Incidence rate of COVID-associated mortality in residents per 1000 person-years†			
Incidence of all-cause mortality in residents per 1000 person-years†			
Incidence rate of SARS-CoV-2 infections in residents per 1000 person-years†			
Incidence rate of home-level outbreaks per 1000 days†			
Incidence rate of care home closures due to outbreaks per 1000 days†			
	**Mean; Median (IQR, range)**	**Mean; Median (IQR, range)**	**Ratio of means* (95% CI)**
Duration of outbreaks, days			
	**Marginal mean; Median (IQR, range)‡**	**Marginal mean; Median (IQR, range)‡**	**aOR* (95% CI, *P*); marginal difference (95% CI)**
Proportion of staff testing each week (%)			
Prevalence of SARS-CoV-2 among staff who test each week (%)			
Proportion of staff per home off sick each week (%)			
Proportion of all shifts filled by agency staff each week (%)			
Descriptive data	**Mean; Median (IQR, range)**	**Mean; Median (IQR, range)**	
n outbreaks per home†			—
n closures per home†			—
Proportion of staff explicitly opting out of testing each week (%)	—		—
Proportion of staff per home off sick with COVID-19 each week (%)			—

*Adjusted for care home provider, region and size and calendar time. †Descriptive data and statistical model summaries do not include data from first 2 weeks of trial participation at each site. ‡Median, IQR and range given for aggregate values for each site.

Table 2 [reduced version for interim report]: Summary and analyses of primary and secondary outcomes

	Trial arm		
	Control	Intervention	**Intervention vs control**
	**Median (IQR, range)**	**Median (IQR, range)**	
Weeks of participation per care home			—
Primary outcome	**Overall [event count]; Median (IQR, range) per home**	**Overall [event count]; Median (IQR, range) per home**	**IRR* (95% CI, *P*)**
Incidence rate of COVID-19 associated hospital admissions per 1000 person-years [site-supplied data]†			
Secondary outcomes	**Overall [event count]; Median (IQR, range) per home**	**Overall [event count]; Median (IQR, range) per home**	**IRR* (95% CI, *P*)**
Incidence rate of COVID-associated mortality in residents per 1000 person-years [based on testing data only]†			
Incidence rate of SARS-CoV-2 infections in residents per 1000 person-years†			
Composite incidence rate of COVID-19 associated hospital admissions and mortality per 1000 person-years†			
	**Overall mean; Median (IQR, range)‡**	**Overall mean; Median (IQR, range)‡**	**aOR* (95% CI, *P*)**
Proportion of staff testing each week (%)			
Prevalence of SARS-CoV-2 among staff who test each week (%)			
Proportion of staff per home off sick each week (%)			
Proportion of all shifts filled by agency staff each week (%)			
Proportion of staff explicitly opting out of testing each week (%)	—		—

*Adjusted for care home provider, region and size and calendar time. †Descriptive data and statistical model summaries do not include data from first 2 weeks of trial participation at each site. ‡Median, IQR and range given for aggregate values for each site.

Graphs

Graph 1: Flow diagram of study site enrolment and intervention implementation, following CONSORT guidelines for cluster trials[3]

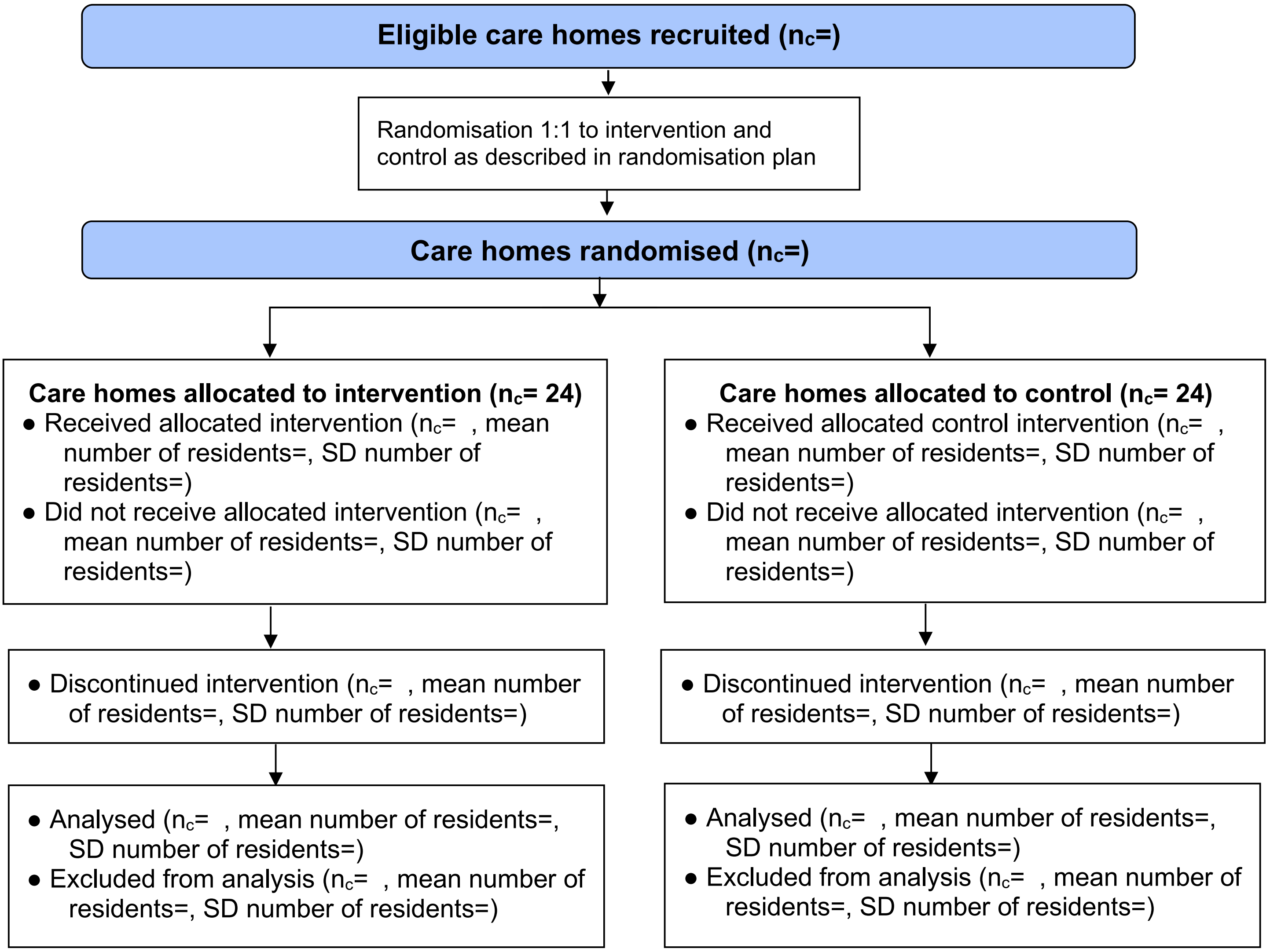

12 REFERENCES

1. Yu H, Li F, Gallis JA, Turner EL. **cvcrand: A Package for Covariate-constrained Randomization and the Clustered Permutation Test for Cluster Randomized Trials**. *R Journal* 2019; 9(2).
2. Kahan BC, Rushton H, Morris TP, Daniel RM. **A comparison of methods to adjust for continuous covariates in the analysis of randomised trials**. *BMC Medical Research Methodology* 2016; 16(1):42.
3. Moher D, Hopewell S, Schulz KF, Montori V, Gøtzsche PC, Devereaux PJ, et al. **CONSORT 2010 Explanation and Elaboration: updated guidelines for reporting parallel group randomised trials**. *Journal of Clinical Epidemiology* 2010; 63(8):e1-e37.
