# Supplementary material for: VIVALDI-CT shaping care home COVID-19 testing policy: A pragmatic cluster randomised controlled trial of asymptomatic testing compared to standard care in care home staff
Source: PLoS One. 2025 Jul 2;20(7):e0324908. doi: 10.1371/journal.pone.0324908 (PMC12221029; doi:10.1371/journal.pone.0324908)
Supplement: Attachment — Submitted filename: COVID-19 VIVALDI TRIAL REVIEW.docx [file pone.0324908.s010.docx]

**PLOS ONE PEER REVIEW**

TITLE: VIVALDI-CT Shaping care home COVID-19 testing policy: A pragmatic cluster

randomised controlled trial of asymptomatic testing compared to standard care in care

home staff.

PONE-D-24-37992

**SUMMARY AND OVERALL IMPRESSION**

This is a methodologically novel study by Stirrup et al., primarily aimed at exploring the difference between future hospital admissions among the elderly who were asymptomatic vs symptomatic or contacts (per standard testing guidelines) for COVID-19 through testing, in order to direct national policy. The use of a Randomized Control Superiority Trial in this context was a perfect design. The study procedures and processes were clearly described and explained

Older people have been found to have a higher incidence of asymptomatic COVID-19, which also carried a significant risk of transmission. This study was therefore in keeping with the findings and recommendations by Barboza et al (2021). Unfortunately, the low recruitment and primary outcome rendered the study statistically less powered at an early stage due to the rapidly changing epidemiological trend. The authors have also been able to clearly outline the major limitations of the study rather than cooking results and making significance out of nothing.

**MAJOR ISSUES**

There was no major issue to warrant correction to the best of my knowledge, expertise and understanding of this study.

**MINOR ISSUES**

For the first sentence of the first paragraph under results, line 270 on page 21, I think the sentence should begin with a word, not a figure (i.e. Eighty-five (85) care homes…..)

**MISCELLANEOUS COMMENTS**

I hereby declare that I do not have any competing interest as far as this study is concerned.
